# Measuring and assessing the competencies of preceptors in health professions: a systematic scoping review

**DOI:** 10.1186/s12909-020-02082-9

**Published:** 2020-05-24

**Authors:** Andrew D. Bartlett, Irene S. Um, Edward J. Luca, Ines Krass, Carl R. Schneider

**Affiliations:** 1 School of Pharmacy, Faculty of Medicine and Health, The University of Sydney, Sydney, NSW 2006 Australia; 2 University Library, The University of Sydney, Sydney, Australia

**Keywords:** Preceptorship, Preceptor, Assessment, Competency

## Abstract

**Background:**

In healthcare, preceptors act as a role model and supervisor, thereby facilitating the socialisation and development of the preceptee into a professional fit to practice. To ensure a consistent approach to every preceptorship experience, preceptor competencies should be measured or assessed to ensure that the desired outcomes are achieved. Defining these would ensure quality management and could inform development of an preceptor competency framework.

This review aimed to evaluate the evidence for preceptor competencies and assessment in health professions.

**Methods:**

This study followed the PRISMA ScR scoping review guidelines. A database search was conducted in Embase, Medline, CINAHL and IPA in 2019. Articles were included if they defined criteria for competency, measured or assessed competency, or described performance indicators of preceptors. A modified GRADE CERQual approach and CASP quality assessment were used to appraise identified competencies, performance indicators and confidence in evidence.

**Results:**

Forty one studies identified 17 evidence-based competencies, of which 11 had an associated performance indicator. The competency of preceptors was most commonly measured using a preceptee completed survey (moderate to high confidence as per CERQual), followed by preceptor self-assessment, and peer-assessment. Preceptee outcomes as a measure of preceptor performance had good but limited evidence.

**Conclusions:**

Competencies with defined performance indicators allow for effective measurement and may be modifiable with training. To measure preceptor competency, the preceptor perspective, as well as peer and preceptee assessment is recommended. These findings can provide the basis for a common preceptor competency framework in health professions.

## Background

Preceptorship may be defined as the formal arrangement, situated within a clinically related setting, between a practicing health professional (the preceptor) and a graduate or student (the preceptee). The preceptor acts as a role model, supervises, provides guidance, learning experiences, and facilitates the socialisation and development of the preceptee into a competent professional, fit for practice during the taught curriculum and pre-registration [1–3]. In some countries, professional bodies mandate a period of supervised practice or an internship prior to full registration [4]. The nature of the preceptor’s role will differ depending on the scope of supervision, which can range from a short-term clinical placement within an undergraduate/postgraduate curriculum to a long-term pre-registration internship [5]. A good placement or internship experience lays a solid foundation for development of professionalism [6] throughout a practitioner’s career. Developing and supporting preceptors also leads to improvements in retention and satisfaction of new graduates [7].

Competencies comprise a combination of knowledge, skills, abilities or attributes[66] . Multiple preceptor competencies have been articulated in the literature such as effective communication skills and being a role model practitioner [8–11]. However, the evidence for identified competencies have yet to be evaluated. To ensure a consistent approach to every preceptorship experience, it then follows that competencies are measured or assessed to ensure that delivery is of a standard that achieves the desired outcomes. Assessment allows for setting a benchmark for comparison as well as for measuring the effect of change over time, eg the effect of an educational intervention such as a training program. Defining the standards that preceptors should strive to attain, as well as methods of assessment, could inform development of a preceptor competency framework and a standard by which preceptors may be measured.

### Aim

The aim of this review was to evaluate the evidence for preceptor competencies and assessment in health professions. The objectives of this review were to:

(i) Evaluate evidence for competencies or performance indicators of preceptors in health professions;
(ii) Describe implemented methods of measurement and assessment of competency;

### Research question

What is the evidence for preceptor competencies in health professions and to how are they assessed?

### Operational definitions

- Preceptorship: Preceptorship is the formal arrangement between a practicing health professional (the preceptor) and a graduate or student (the preceptee). Within a clinically-related setting, the preceptor supervises, provides guidance and facilitates the socialisation and development of the preceptee into a competent professional fit for practice [1–3].
- Assess: To consider (give careful thought to) someone or something and make a judgement about them or it [12].
- Measure: to determine magnitude or quantity based on a standard [13].
- Rating: a classification based on assessment of quality, standard or performance [14].

## Methods

The literature was comprehensively searched using the following databases: Embase, Medline, Cumulative Index of Nursing and Allied Health Literature (CINAHL) and International Pharmaceutical Abstracts (IPA). A search strategy was developed via consensus with all authors and then applied to each database by the primary author (AB) on 19th June 2019 with no date limitation applied. The following PCC (population, concept, and context) strategy was developed a priori. The study population included medicine, nursing, pharmacy or other allied health practitioners. The concept of preceptor was captured using the terms clinical teacher, clinical educator, clinical supervision, preceptor, preceptorship, tutor or clerkship. The context of professional competence incorporated evaluation, guideline, framework, education, skill or quality. Each term was grouped with the boolean operator “OR”, and each concept with the operator “AND”. The search was restricted to peer-reviewed journal articles and those published in English. The search strategy used for Medline is presented in Additional file 1.

### Selection criteria

The inclusion and exclusion criteria were developed in an iterative fashion as described by Arksey and O’Malley [15] as more familiarity with the literature was gained (Table 1). A systematic approach was taken based on the PRISMA-ScR (Preferred Reporting Items for Systematic Scoping Reviews) guidelines [16]. Search results were collated in the reference management program EndNote, then de-duplicated. All titles, abstracts, and full-text articles were screened by the primary author (AB). A random sample of 10% of citations were assessed for eligibility by two additional authors (IU and CS), with consensus agreement being reached. Reference lists were hand searched to identify any additional articles that may fit the eligibility criteria.

**Table 1:** Eligibility criteria

Inclusion criteria	Exclusion criteria
Primary research articles, synthesised findings from literature review, or expert opinion that: - define criteria for the competency of preceptors - measure or assess the competency of preceptors - describe performance indicators of preceptors	Non-English language Studies focused on preceptee competence Reviews of clinical programs Evaluation of preceptor development programs Conference abstracts Full text unable to be obtained Unsupported opinion papers

### Data extraction

Data were extracted and analysed by the primary author (AB), using a standardised data extraction form containing a predefined set of items. Items included study characteristics (e.g. author; year; setting; health discipline; type of study/study design; sample size); mode of measurement, measurement tool and scale; competency criteria; results; reliability/validity. The form was pilot tested with three articles, and discrepant items were clarified and resolved by discussion.

### Risk of bias appraisal

As the included articles in this review had mixed study designs, two critical appraisal tools were used, the Critical Appraisal Skills Program (CASP) Cohort checklist [17] and the Qualitative checklist [18]. The primary author (AB) allocated each article to either checklist, depending on the study design, and evaluated all included articles. A random article from each checklist was independently evaluated by two authors (IU and CS). Any discrepancies identified were resolved by discussion and consensus agreement. A traffic light system was devised to visually describe the articles in terms of each of the CASP criteria; that is, addressed (green), not addressed (red), or unclear (orange).

### Assessment of confidence

To assess the level of confidence in the findings, an approach based on the GRADE CERQual (CERQual) framework was followed [19]. CERQual is an approach that is usually applied to synthesize qualitative findings and to assess confidence in the evidence. While studies in the review were a mix of qualitative and quantitative evidence, the narrative nature of the findings warranted the use of CERQual. A conservative evidence synthesis approach was adopted with synthesis performed by AB and a random 10% selection independently reviewed by CS and IU. CERQual has four criteria (methodological limitations, coherence concerns, adequacy concerns and relevance concerns) against which the included articles were assessed leading to an overall assessment of confidence, described below.

- Methodological limitations were assessed by looking at aspects of each contributing study that may reduce the confidence in the finding [20].
- Coherence refers to the extent to which contributing studies fit with the finding in a convincing way. Studies that contain contradictory results to the other contributors would be seen to reduce the confidence in the finding [21].
- Assessing adequacy involves making a judgement on the quantity of data along with the quality or richness of the information gained [22].
- The confidence in the relevance of the papers contributing to the finding was a matter of examining the setting, context, perspective and phenomenon of interest [23].

After these assessments were made, they were considered as a whole to determine confidence in the evidence for the finding. These were then graded on a scale from low to high confidence. All findings were synthesised narratively.

## Results

### Study selection

**Fig. 1:**
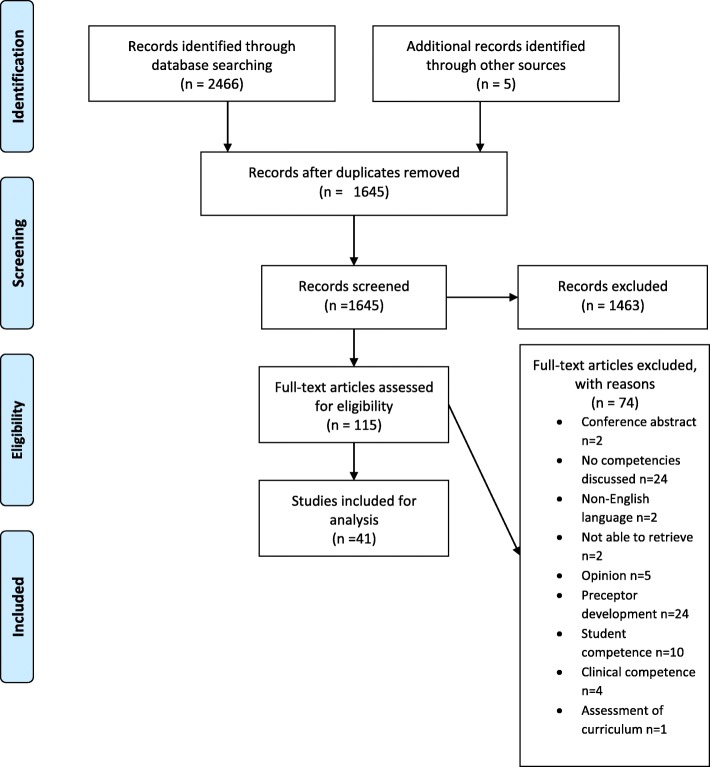
PRISMA flow diagram of process to identify eligible articles ^63^

The literature search retrieved a total of 1642 citations after removing duplicates. Screening for eligibility based on titles excluded 1463 papers, leaving 179 papers for review of abstracts. A further 69 papers were excluded after reviewing abstracts, with 110 papers carried forward for full text review. Careful screening provided 36 papers fitting the selection criteria. Hand searching found an additional 5 references, resulting in a total of 41 [8–11, 24–60] articles to be included for review (Fig. 1).

### Study characteristics

Of the 41 included articles, 26 were conducted in the United States [9, 10, 25, 26, 28–31, 33–36, 38–40, 44–48, 50–52, 56, 59], three in Canada [8, 11, 54], two in Taiwan [42, 43], two in Iran [27], and one each in Thailand [57], Ethiopia [53],Saudi [24], Brazil [37], Australia [49], Sweden [58], Belgium [55] and the United Kingdom [32]. Seventeen were based on preceptorship/education in medicine [10, 31, 32, 35, 44, 46, 50–52, 54–56, 58–60], twelve in pharmacy [9, 11, 24–26, 30, 33, 34, 36, 38, 53, 57], and eleven in nursing [8, 27, 28, 37, 40–43, 47–49] and one in dentistry [45]. There were twelve quantitative studies [25, 26, 30, 33, 35, 39, 46, 47, 49, 53, 57], ten qualitative studies [29, 34, 37, 41, 42, 44, 45, 48, 58, 59], three mixed methods [43, 50, 51], four descriptive papers that did not report results [36, 38, 40, 56], five papers concerned with validity testing [28, 31, 52, 54, 55] and seven papers describing consensus building, three with Delphi approaches [10, 29, 32] and four with expert opinion based on literature review and qualitative synthesis [8, 9, 11, 60]. Data were extracted and are presented in Additional file 2.

### Competencies and methods of assessment of preceptors

Seventeen competencies with associated methods of assessment were identified, as outlined in Table 2. The methods used to identify competencies of preceptors included expert opinion based on literature reviews and qualitative synthesis [8, 9, 11, 60, 61], Delphi approaches [10, 29, 32], and qualitative studies examining the qualities of preceptors that preceptees value most [27, 37, 42, 44, 45, 51]. Fifteen studies identified competencies, but assessment had not been implemented or reported [8–11, 27, 29, 32, 37, 38, 41, 45, 48, 56, 58, 60], with only four studies detailing performance indicators for the competencies described [8–11].

**Table 2:** Preceptor competency and assessment in health professions

Competency with identified measures of performance	GRADE CERQual Confidence in evidence	Assessment measure	Setting
-Effective communication skills [8–11, 24, 25, 29–33, 42, 45, 47–49, 53, 57, 59, 60]	Moderate to high	Peer observed practice [30, 33, 42, 59] Peer observed simulation [31]	Medicine [31, 59] Nursing [42] Pharmacy [30, 33]
		Preceptor self-evaluation survey [26, 43, 53, 57]	Nursing [43] Pharmacy [26, 53, 57]
		Preceptee survey [8, 26, 33, 53, 57]	Nursing [8] Pharmacy [26, 33, 53, 57]
-Role model practitioner [8–10, 24, 25, 29, 30, 32, 33, 41–44, 46, 48, 49, 58, 59]	Moderate to high	Peer observed practice [30, 33, 36, 42, 59]	Medicine [59] Nursing [42] Pharmacy [30, 33, 36]
		Preceptor self-evaluation survey [26, 36, 43]	Nursing [43] Pharmacy [26, 36]
		Preceptee survey [8, 26, 36, 46]	Medicine [46] Nursing [8] Pharmacy [26, 36]
-Adapts to the learning needs of students [10, 11, 30, 37, 44, 47, 58, 59]	Moderate to high	Peer observed practice [30, 31, 42, 59] Peer observed simulation [55]	Medicine [31, 55, 59] Nursing [42] Pharmacy [30]
-Commitment to excellence in teaching [8, 9, 11, 29, 30, 32, 42–44]	Moderate	Peer observed practice [30, 42]	Nursing [42] Pharmacy [30]
		Preceptor self-evaluation survey [43]	Nursing [43]
		Preceptee survey [8]	Nursing [8]
-Demonstrates respect for the learner [42, 46, 47, 59]	Moderate	Peer observed practice [42, 59]	Medicine [59] Nursing [42]
		Preceptee survey [46, 54]	Medicine [46, 54]
Demonstrate reflective practice [8, 10, 11, 32]	Moderate	Preceptee survey [8]	Nursing [8]
Effective provision of feedback [9–11, 26, 32, 36, 42, 52, 55]	Moderate	Peer observed practice [36, 42] Peer observed simulation [55]	Nursing [42] Medicine [55] Pharmacy [36]
		Preceptee survey [26, 36]	Pharmacy [26, 36]
		Preceptor self-evaluation survey [26, 36]	Pharmacy [26, 36]
Demonstrate reflective practice [10, 11, 29, 32]	Moderate	Peer observed practice [42]	Nursing [42]
		Preceptee survey [8]	Nursing [8]
-Facilitate critical thinking, problem solving and decision-making development [8, 11, 30, 32, 41, 53, 55, 57, 59]	Moderate	Peer observed practice [30, 59] Peer observed simulation [55]	Medicine [55, 59] Pharmacy [30]
		Preceptor self-evaluation survey [53]	Pharmacy [53]
		Preceptee survey [8, 53, 57]	Nursing [8] Pharmacy [53, 57]
-Encourage self-directed learning [9, 52, 54, 56]	Moderate	Preceptee survey [52, 54]	Medicine [52, 54]
-Leadership and management skills [9, 25]	Low-moderate		
**Skills of effective preceptors without indicators of performance identified.**	**GRADE CERQual Confidence in evidence**	**Assessment measure**	**Setting**
-Organised and ability to prioritize [32, 33, 42, 43]	Moderate	Peer observed practice [33, 42]	Nursing [42] Pharmacy [33]
		Preceptor self-evaluation survey [43]	Nursing [43]
-Empathetic [27, 30, 32, 41, 47]	Moderate	Peer observed practice [30]	Pharmacy [30]
-Ethical [31, 32, 42]	Moderate	Peer observed practice [31, 42]	Nursing [42] Medicine [31]
-Approachable and flexible [26, 30, 42, 45–47]	Moderate	Peer observed practice [30, 42]	Nursing [42] Pharmacy [30]
		Preceptee survey [26, 46]	Medicine [46] Pharmacy [26]
-Enthusiasm for teaching Preceptees [32, 33, 36, 42, 43]	Moderate	Peer observed practice [33, 36, 42]	Nursing [42] Pharmacy [33, 36]
		Preceptor self-evaluation survey [36, 43]	Nursing [43] Pharmacy [36]
		Preceptee survey [36]	Pharmacy [36]
-Open to receiving feedback [26, 32, 36, 46]	Low	Peer observed practice [36]	Pharmacy [36]
		Preceptor self-evaluation survey [26]	Pharmacy [26]
		Preceptee survey [26, 46]	Medicine [46] Pharmacy [26]

The competency of preceptors was measured in four ways. Most commonly, and with moderate to high confidence as per CERQual, preceptees used a survey instrument to assess the competency of their preceptor [26, 30, 46, 53, 57]. Preceptee assessment was also combined with a preceptor self-assessment instrument which allowed for comparison between preceptors’ and preceptees’ perceptions of their experience working together [36, 53, 57]. Preceptors were shown to overestimate their abilities when self-evaluating, compared to preceptee evaluations (CERQual assessment: low confidence). Another method used was an assessment of preceptors by peers or faculty using a survey instrument [30] or by direct observation of interactions with preceptees [31, 33, 59]. While this approach provided greater specificity and detail [50], it was reported as more difficult to implement on a large scale, due to time and cost constraints, as well as lower preceptor acceptance [30]. The fourth approach was associating preceptees’ examination performance with the ‘quality’ of teaching as rated by preceptees via subjective measurement [39]. While the strongest evidence exists for preceptee evaluation of preceptors; there is moderate confidence in evidence for self-assessment and peer evaluation, the confidence is lowered by the limited amount of evidence. Although there was good correlation between preceptee evaluations of preceptors and preceptors’ self-assessment; preceptors overestimated their effectiveness as communicators [53] and their ability to provide feedback [53, 57]. Measurement of preceptee outcomes had good but limited evidence (low confidence) [39]. Only one study linked the quality of the preceptor with a preceptee outcome; preceptees with a perceived higher preceptor quality performed better in their exams [39]. Table 2 describes the methods of assessment, and confidence in evidence. The full CERQual evaluation is presented inpresented in Additional file 3.

### Quality appraisal

The CASP Cohort checklist was used for the quantitative and validity testing papers, and the CASP Qualitative checklist was used for qualitative studies, descriptive studies and consensus building based on literature review and qualitative synthesis. The results of the CASP assessments tabled with a traffic light legend can be found in Additional file 4. Very few studies received green ratings across all categories. In the CASP Qualitative assessment, only two papers were green in all catagories [42, 44]. Most commonly, articles did not contain enough information to make an assessment on the relationship between participants and researchers, ethical considerations, and data analysis. For the CASP Cohort assessment, there were no randomised controlled trials, and no studies received green ratings on all categories. Most commonly, articles did not have enough information regarding follow up of participants, or the length of follow-up of participants. Many articles did not have enough information to ascertain whether confounding factors were considered in the study design.

## Discussion

This systematic scoping review of the literature and evaluation of the quality of evidence using GRADE CERQual, informed the development of a 17-item evidence-based set of preceptor competencies and corresponding methods of assessment that is applicable to a diverse range of health professions. This review did not find evidence for significant differences for requisite preceptor competencies across health professions. The identification of the minimum level of performance at which a health practitioner may be deemed a ‘competent’ preceptor requires calibration. These competencies and methods of assessment may form the basis for a competency framework and be applied to recognise preceptors working at an advanced level of practice, thereby enabling a system of quality management and oversight.

In assessing the confidence in evidence for these findings using CERQual, it was apparent that 11 of the identified competencies have defined performance indicators that may allow for effective measurement of competence, while six could be described as attributes. Attributes such as being ethical, enthusiastic, or empathetic, were not associated with performance indicators and would therefore to be less conducive to measurement and standardisation. Without adequate measurement, discerning the effect of any potential intervention, such as training is problematic. Interestingly, the competencies or attributes without performance indicators, such as empathy, could be considered as intrinsic to the individual preceptor. Intrinsic traits have been identified as being difficult to modify through training but may develop with personal reflection and maturity [60]. Sutkin and colleagues conclude that affective or non-cognitive characteristics are of greater importance than the skill based cognitive abilities in making a “truly great” preceptor. A way forward is proposed by Davis (1989) who recommends that preceptors model empathy as an extrinsic behaviour in order to facilitate preceptee development via professional socialisation [62]. By modelling intrinsic affective traits as behaviours, measurement is thereby possible.

A disconnect between the competencies and the mode of measurement of some competencies was identified. For instance, adapting to the learning needs of preceptees had evidence for assessment by peer observation in both simulation and practice environments. This would seem to be a competency that lends itself to evaluation from the perspective of the preceptee, however, in the literature there was no evidence for this mode of measurement. Likewise, demonstrating reflective practice had evidence for assessment via a preceptee survey, whereas self-reflection as a mode of measurement would seem logical, but again, evidence was not apparent. A recommendation is to consider alignment of the mode of measurement with the competency being assessed in a consistent manner across all competencies in the framework.

There was a lack of evidence to demonstrate a relationship between competencies or attributes of preceptors and preceptee outcomes. According to Bigg’s framework of constructive alignment, learning outcomes should be clearly outlined at the beginning, then learning activities and assessment aligned, so that the level to which those outcomes have been achieved can be measured [63]. In the context of preceptorship, this framework could be applied. Preceptor competencies would be the outcome to be measured (and potentially also the preceptees’ exam performance) against a defined standard, and preceptor development constitutes the learning activities. The competencies being measured and the mode of measurement also needs to be aligned. Gill (2004) notes that the ultimate goal would be to link preceptor performance and preceptee learning [61]. Whilst Griffith (2000) linked preceptee performance in an exam with perceived preceptor quality, the competencies that led to those preceptors being rated highly were not detailed [39].

An additional consideration in constructive alignment would be to align assessment with assessors who are well placed to provide the assessment. Brookfield describes four lenses through which teachers can view their practice from different perspectives as a tool for critical reflection and ultimately to perform more confidently and at a higher level [64]. A tripartite preceptorship model with preceptor, preceptee and faculty being in partnership with assessment from all perspectives, along with some theoretical training, would fit this model. At a minimum, all preceptors should undertake preceptor development training and evaluation by their preceptees. A portfolio of evidence could then comprise of assessment from all partners. Registration bodies who conduct examinations on behalf of professional boards could provide feedback to preceptors on preceptee performance, preceptees could provide evaluations of the preceptor at various timepoints, and preceptors could include a reflective self-evaluation of their performance. Peer assessment would be more expensive to deploy on a large scale and may be less acceptable and convenient, however, judicious use of peer assessment would provide a high degree of detail and specificity (CERQual assessment: high confidence. Finally, the validity and reliability of the assessment method also needs to be considered. If this framework were to be applied to credential preceptors at an advanced level of practice, peer observation and evaluation should also be included as part of a portfolio of evidence built over time. Finally, the validity and reliability of the assessment method also needs to be considered. If this framework were to be applied to credential preceptors at an advanced level of practice, peer observation and evaluation should also be included as part of a portfolio of evidence built over time.

The strengths of this scoping review are that a rigorous, standardised approach of CERQual was used to assess the confidence in the evidence. This provides credibility to the competencies and methods of assessment identified from the literature search. The nature of the scoping review process allowed for the inclusion of papers of various study design such as validity testing and qualitative studies. A conservative synthesis approach was adopted to facilitate inclusivity of the language used to describe competencies across the literature. Further rationalisation of the identified competencies may be possible. The review followed the PRISMA ScR guidelines and an iterative process was maintained between authors. Limitations of the review are that title and abstract screening, data extraction and evidence assessment were primarily carried out by the primary author, although a random selection of results for all stages were assessed by two additional authors, with points of difference discussed to achieve consensus. An additional limitation was the preponderance of studies from a single country (USA), resulting in a potential risk to generalisability of findings.

### Implications for future

This review has synthesised a common set of preceptor competencies across health professions. Additional unique preceptor competencies for individual health professions may need to be considered. The relationship between measuring the performance of preceptors and effective outcomes of preceptees has yet to be determined and requires further investigation. It would be worthwhile to examine which of the competencies identified are most relevant to the outcomes for graduates. Retention in the workforce, professional satisfaction, and career progression are all outcomes that may indicate that the model of preceptorship is successful, but this requires evaluation. In addition, the minimum level of preceptor competence has not been determined. A consensus approach, such as the Delphi method [65], could be used to improve confidence in the identified competencies and determine the minimum standard against which preceptors should be assessed. Future primary studies with the aim to identify variation in preceptor competencies between professions are required.

## Conclusion

A standardised, evidence-based set of preceptor competencies and accompanying methods of assessment has been identified across health professions. Most competencies have an associated performance indicator which allow for effective measurement, while others are more intrinsic to the individual resulting in measurement difficulty. Further research is required to identify the minimum standard of performance that is necessary, as well as to identify the factors that have the greatest influence on the outcomes for preceptees.

## Supplementary information

**Additional file 1.** Appendix 1 – Example search strategy using Medline.

**Additional file 2.** Appendix 2 – Data extraction table.

**Additional file 3.** Appendix 3 – GRADE CERQual evaluation table.

**Additional file 4.** Appendix 4 Risk of Bias appraisal.

## Data Availability

All data generated or analysed during this study are included in this published article [and its supplementary information files].

## Abbreviations

PRISMA Scr: Preferred Reporting Items for Systematic reviews and Meta-Analyses extension for Scoping Reviews.
GRADE: Grading of Recommendations Assessment, Development, and Evaluation.
CERQual: Confidence in the Evidence from Reviews of Qualitative research.
CASP: Critical Appraisal Skills Programme
AB: Andrew Bartlett
IU: Irene Um
CS: Carl Schneider
IK: Ines Krass
EL: Edward Luca
