# Supplementary material for: Measuring and assessing the competencies of preceptors in health professions: a systematic scoping review
Source: BMC Med Educ. 2020 May 24;20:165. doi: 10.1186/s12909-020-02082-9 (PMC7247189; doi:10.1186/s12909-020-02082-9)
Supplement: Supplementary file 1 — Additional file 1. Appendix 1 – Example search strategy using Medline. [file 12909_2020_2082_MOESM1_ESM.docx]

Appendix 1 – Example search strategy using Medline

| Ovid MEDLINE(R) <1946 to June Week 2 2019> | | | |
| --- | --- | --- | --- |
| **#** | **Search Statement** | **Results** | **Annotation** |
| 1 | exp education, continuing/ or exp education, dental/ or exp education, graduate/ or exp education, medical/ or exp education, nursing/ or exp education, pharmacy/ or education, public health professional/ | 274171 |  |
| 2 | exp Preceptorship/ | 4864 |  |
| 3 | preceptor*.mp. | 6249 |  |
| 4 | Professional Competence/ | 23636 |  |
| 5 | (preceptor* adj4 (evaluat* or guideline* or competenc* or framework* or educat* or skill* or qualit*)).mp. | 706 |  |
| 6 | clinical supervision.mp. | 1236 |  |
| 7 | clinical supervisor.mp. | 90 |  |
| 8 | clinical educator.mp. | 81 |  |
| 9 | clinical teacher.mp. | 229 |  |
| 10 | exp Allied Health Personnel/ | 48001 |  |
| 11 | exp Physical Therapists/ | 1482 |  |
| 12 | exp Occupational Therapists/ | 179 |  |
| 13 | 1 or 10 or 11 or 12 | 318996 |  |
| 14 | 2 or 3 or 6 or 7 or 8 or 9 | 7753 |  |
| 15 | 4 or 5 | 24298 |  |
| 16 | 13 and 14 and 15 | 664 |  |
