# Supplementary material for: Measuring and assessing the competencies of preceptors in health professions: a systematic scoping review
Source: BMC Med Educ. 2020 May 24;20:165. doi: 10.1186/s12909-020-02082-9 (PMC7247189; doi:10.1186/s12909-020-02082-9)
Supplement: Supplementary file 2 — Additional file 2. Appendix 2 – Data extraction table. [file 12909_2020_2082_MOESM2_ESM.docx]

Appendix 2 – Data extraction table

|  | **Main Author** | **Year** | **Setting** | **Discipline** | **Type of study** | **Sample size** | **Mode of measurement**  **Measurement tool and scale** | **Competencies criteria** | **Results** | **Reliability/Validity** |
| --- | --- | --- | --- | --- | --- | --- | --- | --- | --- | --- |
| 1 | Boland | 2014 | America: Hospital | Pharmacy | single-centre, Prospective cohort; post-survey | Preceptors (n=8) | **Student evaluation** (n=23)  And **Preceptor self-evaluation** (n=8)  Survey- Likert  Always-usually-often-Seldom | **-Student assessment of preceptor**  1) Demonstrate knowledge of therapeutics.  2) Demonstrate ability to complete literature searches and apply evidence.  3)Professional role model  4) Model appropriate relationships with other health professionals.  5)Demonstrate effective patient communication skills  6) Regular feedback on strengths and areas of improvement.  7) Available and approachable.  8) Provide a good learning experience.  9) Encourage initiative in patient care and learning opportunities  10) Would student recommend this preceptor.  **Self Evaluation by preceptor**  1)I was able to teach the student about therapeutics, literature searches and evidence based medicine above student level.  2)I was a good role model  3) My communication was constructive and positive.  4) I was able to motivate student ot learn.  5) The students assessment of my strengths was what I expected.  6) I will use students comments to build upon my strengths.  7)Students suggestions for improvement were what I expected  8)I will use the suggestions for improvement to help me grow as a preceptor.  9)Student feedback will help me become a stronger preceptor.  10)I am and effective preceptor.  11)Overall the student evaluation is what I expected. | Students responses and resident response showed good correlation. Differences found related to instructing and facilitating | Internal Validity  Subjective  Small sample size,  Single centre. Survey was not validated.  Process issues e.g. incorrect links for so some students judged incorrect preceptor |
| 2 | Bradley | 2015 | America: Hospital | Nursing | Multisite, Content Validity Methodological study. | Nurse experts rating usefulness N=12  Test-Re-test preceptors n=9 preceptees n=10  Internal consistency Preceptors n=66, preceptees n=43 | Validity established using 4 point likert scale of 4=very useful and 1= not useful)  The instrument: 4 point likert scale (4= strongly agree and 1= strongly disagree) | Assessed learning needs  Built on previous experience  Collaborated to design leaning goals  Taught according to a preferred learning style  Explain though processes to teach problem solving  Asked open ended questions to facilitate thinking  Structure learning from simple to complex  Provide meaningful feedback  Modelled debriefing  Role modelled professional behaviour | 25 of the 28 items were identified as useful | Internal consistency and test-retest both had strong scores showing that the instrument was a reliable method of assessment   - Recruitment strategy was the investigator recruiting through group or individual meeting- not 3^rd^ party |
| 3 | Childs-Kean | 2016 | America-Hospital | Pharmacy | Peer evaluation of preceptor and assessment of the survey | Preceptors assessed N=21  Tool Evaluations completed N=17 | **Student assessment.**  Likert scale  From Strongly disagree to strongly agree and not applicable | 1. The preceptor spent time orienting the student and describing goals and expectations 2. The preceptor spent time observing the student and assessing performance 3. The preceptor provided constructive feedback on a regular basis 4. The preceptor encouraged exploration and application of problem solving skills 5. The preceptor arranged the necessary learning opportunities to meet learning objectives 6. The preceptor communicated subject matter effectively by giving explanations, asking questions and giving instruction for further learning 7. The preceptor modelled, coached performance, and facilitated independent work as appropriate. 8. The preceptor created a stimulating learning environment by being supportive, enthusiastic, friendly and accessible 9. The preceptor demonstrated concern for the student | The tool was found to be easily to administer. Some found there was not enough time to address all question, particularly question 1 | Not validated with multiple uses nor compared to other evaluations.  Small number of tools completed.  Possible Hawthorn effect- changing behaviour knowing you are being observed. |
| 4 | Cotton | 2009 | UK- General practice | Medicine | Workshop developing criteria followed by Online Delphi questionnaire and stakeholder enquiry | Workshop participants N=44  Delphi rnd 1 N=52 invited n=44 responded  Delphi rnd 2 N=62 invited 44 responded.  Stakeholder N=62 | Criteria rated as Essential, desirable, unnecessary | Tutor characteristics Enthusiasm/commitment to teaching  Awareness of importance of role modelling  Attends teacher development courses on an ongoing basis Able to give constructive feedback  Able to undertake formative assessment  Awareness of ethical standards relating to teaching Method of assessment are made clear to students  Able to ensure protected time for teaching Willing to be evaluated/appraised as a teacher Willing to reflect/act on student feedback Evidence of competent communication skills No serious upheld complaint  Willing to take responsibility for teaching schedule Willing to prepare practice colleagues Aware of curriculum/objectives of teaching Aware of teacher/student assessment processes Demonstrates high expectations of students Commitment to the pastoral care of students  Understanding of basic educational theory/principles Commitment to demonstrating reflective practice Acknowledges students’ needs  Willing to undergo peer review of teaching Holds Membership of the Royal College of General Practitioners | 58 criteria in four domains   1. Physical environment 2. Learning environment 3. Tutor characteristics 4. Patient involvement   In addition   1. Departmental responsibilities | Strengths- wide range of participants, quant and Qual data, consensus created iteratively.  Weakness- long list of criteria difficult to discriminate, uncertainty over reaching theoretical saturation due to small number of stakeholders.  Student participants from one school only |
| 5 | Cox | 2013 | America: Faculty | Pharmacy | Single site Quantitative evaluation | Peer and student evaluation of preceptors n=8 | **Peer and student evaluation**  : Survey- Formative comment and closed question survey with Likert scale (1=Poor, 2=Fair, 3=Good, 4=Excellent, 5=Outstanding) :Initial evaluation and 7 year follow up | 1. Does the preceptor possess an enthusiasm for teaching? describe  YES or NO. Please  2. Does the preceptor emphasize the importance of the process of problem solving or are they only critical of the students/resident’s knowledge base? (Do they facilitate critical thinking skills and/or facilitate development and application of knowledge?) Please describe  3. 4. 5.  Is the preceptor well organized? (Has daily/weekly schedule that students/residents follow or are they very erratic and/or spontaneous in their activities?). Please describe  Is the preceptor clinically competent? (Not easy to assess but may be able to comment based on rapport with other team members on rounds/clinics). Please describe  Is the preceptor seen as a positive role model for the student/resident? (Is there respect for the preceptor by the student/resident, does the student/resident look up to the preceptor, seek out advice or ask many questions?) Please describe  6. Does the preceptor exhibit good communication skills? (In other words, are they able to easily convey their thoughts to other health care professionals on rounds/clinics and to their students/residents in patient discussions?) Please describe  7.  Is the preceptor accessible by the student/resident? (Is the student/resident able to contact the preceptor if questions arise?) Please describe  8. Does the preceptor maintain a good balance between supervising students/residents and also allow them to work/learn on their own? (Does preceptor do all the talking or is the student/resident actively involved, does the preceptor make students/residents look up every answer to the questions they ask or do they simply give the answers to all questions that students/residents ask, or is there a good balance?). Please describe  9. Does the preceptor use innovative or creative methods in their teaching? (Have games, trivia days, clinical pearls/drugs of the day?) Please describe | only 5 of 8 remained for the re-test and 3 reported the process had been helpful.  Tendency for higher performing students to rate instructors more highly | Test-Retest reliability.  Participation was voluntary (preceptors interesting in improving their teaching)  Small sample from a single school,  - possible bias.  Reflexivity- participants had been involved in both development and implantation.  Not validated in other setting |
| 6 | De Bueno | 1995 | America: Hospital | Pharmacy | Single site qualitative evaluative | Faculty n=27 | **Performance Based Development** system using audio and video simulation, questions and problem solving exercises.  Qualitative ratings of acceptable, unacceptable or partially acceptable | Assesses critical thinking, interpersonal and technical skills, Ability to meet role expectations, determine collective learning needs and determine readiness for promotion.  **Abilities measured:**   1. Preceptors ability to determine learning needs 2. Ability to identify and manage problems and give relevant rationale for choices 3. Conflict resolution 4. Understanding of policy norms, department values 5. Ability to match learning and practice needs with patient’s needs, also understanding of components of clinical performance based development system 6. Ability to differentiate priorities, needs for intervention and specific actions to manage and event | Participants need to develop conflict resolution skills, develop goal setting strategies and increase assertive communication skills.  As incremental pay increases were being instituted for preceptors, this was an evaluation to see if there was productivity gains being made to offset cost increases | A consultant author reviewed audio and any differences in ratings or interpretation were discussed with the facilitators.  Single site |
| 7 | Elliot | 1989 | America: university | Medicine | Single centre prospective cohort study | Student evaluation N=136 | **Survey on 22 teaching behaviours and overall** **performance** - 5 point likert scale (no other details provided) | Learning Climate, feedback behaviour, encouraging independent study, reinforcing skills, ability to assess ability to analyse and apply knowledge | 2 performance dimensions identified as significant – learning climate and feedback behaviour | Subjective assessment.  Single site |
| 8 | Elmore | 2014 | America : Hospital | Pharmacy | Single sight program development | Preceptor in training program N=26 | Survey: Likert (5=strongly agree 1=strongly disagree) based on ASHP **preceptor and learning experience evaluation form** | **Self Evaluation:** I am a pharmacy practice role model.  I give feedback on a regular basis.  My feedback helps the resident improve performance.  I am available when the resident needs me.  When possible, I arrange the necessary learning opportunities to meet resident objectives.  I display enthusiasm for teaching.  I give clear explanations.  I ask questions that cause the resident to do their own thinking.  I answer resident questions clearly.  I model, coach, and facilitate independent work as appropriate.  I display interest in the resident.  I display dedication to teaching.  **Resident evaluation:** Was prepared to discuss information with me  Facilitated interaction – i.e. did not lecture  Displayed enthusiasm for teaching Provided clear expectations.  Asked questions that caused me to do my own thinking  Applied information discussed to patient care situations  Answered questions clearly Provided me a list of items to  review that I did not know Displayed interest in me Encouraged me to self-assess Asked for/solicited feedback from me  Gave constructive feedback to me (including ways to improve)  **PEER evaluation** - Was a role model  Provided appropriate time allotted to the topic  Facilitated interaction  Displayed enthusiasm for teaching  Gave clear explanations  Asked questions that caused the resident to do their own thinking  Answered questions clearly  Displayed interest in the resident  Encouraged the resident to self- assess  Asked for/solicited feedback from resident  Gave constructive feedback (including ways to improve) | The results of these surveys are reviewed in a one on one meeting with the program director and individual learning plans are developed and helps to standardise development and assessment of preceptors as well as professional advancement | Descriptive paper of program – no results published |
| 9 | Fuller | 2013 | America : Hospital | Pharmacy | Single site  Descriptive paper on Program development | Preceptors N=45 | Submission of academic and professional record with audit of PE points every 2 years | Preceptor must participate in:  education, postgraduate training, professional experience, improvements in and contributions to pharmacy practice, appointments to drug policy and other committees, recognition by peers as a role model, publications, presentations, manuscript review activity, membership, teaching service, and service in national, state, and local professional associations.  The **four preceptor roles identified-** instructing, modelling, coaching, and facilitating | Descriptive paper- no intervention | Descriptive- no evaluation |
| 10 | Gueorguiva | 2016 | Canada : Hospital | Nursing | Review of previous study used to stimulate change to preceptor program | N/A | N/A | The competencies were synthesized into five core pillars: (a) content knowledge-Indicator->Maintains current professional knowledge and supports learner to seek out new knowledge  Models, supports, and encourages learner to integrate current evidence and patient-centred care to deliver optimal care  (b) facilitation of learning-Indicator->Provides a range of clinical experiences, keeping in mind patient safety and learners’ abilities  Reflects on the effectiveness of one’s teaching strategies  (c) interpersonal and communication skills- Indicator ->Demonstrates openness to different ways of thinking and being, Models problem solving and effective conflict resolution techniques Welcomes dialogue and encourages the learner to ask questions  (d) collaboration- Indicator ->Provides opportunities for the learner to interact with interprofessional team members  Helps learner understand team dynamics and processes  and (e) role modelling and professionalism – Indicator ->Role models and inspires best practices and professional behaviours in others : Recognizes the impact of own behaviour on the learner’s professional development | Indicators were used to help with preceptor selection.  Also used to develop and train preceptors.  Used also as a the basis for evaluation survey of preceptors by preceptees ( not implemented)  :Performance indicators also used as basis for reward and career progression | Methods not outlined in detail – involved literature review an consultation with Clinical education experts |
| 11 | Hartline | 1993 | America : Hospital | Nursing | Development of assessment tool based on literature review | N/A | **Self-evaluation and peer evaluation** using assessment tool and then consultation as part of initial preceptor selection then performance review (weighted scale) | Five themes based on 15 qualifications: Weighted  Nursing process 25%  Interpersonal skills 25%  Leadership skills 10%  Teaching skills 20%  Professional attitudes 10%  Narrative statement allows for scores in a range up to the weighted level | The results are to be used both for selection and identifying areas of development | Qualifications based on literature review and current research ( Not outlined) – participation was not mandatory, existing preceptors were reluctant to participate and some potential preceptors who were identified did not apply. |
| 12 | Hsu | 2006 | Taiwan: Hospital | Nursing | Qualitative study of teaching behaviours | Nurse educators N-10  Students N=10 | Observations Coded and categorised into themes of clinical teaching aims | Concept of Clinical teaching  Themes: teaching aims-task orientated or learner centred  Teacher competence: codes: teacher knowledge, Instructional skills, Planning learning experience, teaching priorities, feedback provision, teaching manner  Teaching commitment: modelling professional identity, giving of self | Teachers were found to be task oriented generally, this meant the development of clinical skills, judgement, problem solving did not have as much time dedicated to them | 2 observers ( one a researcher, other independent) both read and coded transcripts- discussion between observers and expert faculty in clinical teaching reasonable level of authenticity  Small sample.  One |
| 13 | HSU | 2014 | Taiwan: Hospital | Nursing | Mixed methods: interviews, quantitative analysis and literature review | Nurses: Pilot study for validation (n=30)  Test sample (n=289) | Instrument based on Sonthisombats 47 item model with literature review and then validity testing resulting in 53 items- reduced to 31 after factor analysis | 4 Factors identified   1. Student evaluation 2. Goal setting and individual teaching 3. Teaching strategies 4. Demonstration of organised knowledge | Proposal of a self-evaluation tool with good validity was the aim. The study found the tool to have adequate construct validity and reliable internal consistency of validity and was found to therefore be a valid self-measure of preceptor performance | No test re-test but factor analysis showed good construct validity and internal reliability.  Subjective self-rating  Generalizability outside of Taiwan not examined.  Correlation of preceptor self-evaluation and student evaluation not studied |
| 14 | Huggett | 2008 | America :Hospital | Medicine | Qualitative study  Single school, maximum variation of students, preceptors and clinical sites | Students (n=110) | Analysis of 2^nd^ year medical students learning journals to get perspective on preceptor quality | Five attributes of an effective preceptor emerged: (1) Demonstrates professional expertise (2) Actively engages students in learning (3) Creates a positive environment for teaching and learning (4) Demonstrates collegiality and professionalism (5) Discusses career-related topics and concerns. | Students have preconceived ideas on what professionalism means. This expectation influences perceptions of preceptor quality. This has implications for their ideas of professionalism and even future career path | Followed established Qualitative methodologies .Trustworthiness: Investigators discussed all observations and interpretation, discussing until consensus was reached.  Participants from a single cohort from a single school.  Students were not prompted to write about preceptor qualities, but prompting this may have revealed more preceptor attributes. Combining with multiple data sources may improve further studies |
| 15 | Johnson | 2006 | America : Hospital | Medicine | Prospective assessment of medical student evaluations of preceptors – comparison of faculty vs senior residents | Students (n-138) evaluating faculty (n=11) and senior resident (n=13) | **Student evaluation** of 15 teaching behaviour items measured on 5 point likert scale (1 strongly agree, 5 strongly disagree) | Teaching behaviours measured   1. Communicated goals and expectations for sessions 2. Was an appropriate clinical role model 3. Established an inclusive, comfortable environment 4. Gave me confidence in their clinical skills 5. Provided me with constructive feedback 6. Emphasized evidence-based learning 7. Was enthusiastic about teaching 8. Actively involved me in the learning process 9. Presented information at my level 10. of understanding 11. Was enthusiastic about patient care 12. Explained their clinical decision-making process 13. Provided opportunities to improve my clinical skills 14. Treated me with respect 15. Made me feel comfortable admitting knowledge gaps 16. Overall teaching was very effective | Faculty scored higher on 4 behaviours – “acting as an appropriate clinical role  model, emphasizing evidence-based learning, and being  Enthusiastic about teaching and about patient care.”  Residents scored higher on “provided me with opportunities to  improve my clinical skills’’.  The suggestion from the findings is faculty and residents provided different contributions, both of which were of importance and hence the combination of faculty and residents was an ideal combination for the preceptorship experience.  Greater exposure to preceptors also increased the evaluation scores. | Conducted in a single institution with a single cohort of students with preceptors who had volunteered for the role.  Students were required to submit appraisal as part of their course- inconsistent with the voluntary and anonymous statement in methods.   - Small number of preceptors in sample   -Methodological concern |
| 16 | Knisely | 2015 | America: Hospital | Nursing | Quantitative study | Preceptors(n=278)  Students (n= 78) | Web-based questionnaire of importance of 21 effective clinical teaching characteristics. | 1. Clinical competence/judgment 2. Calm during times of stress 3. Ego strength/self-assurance 4. Flexibility 5. Appropriately encourages independence 6. Engenders confidence 7. Motivates students 8. Empathy/respect 9. Evaluation/counselling 10. Enjoys teaching 11. Stimulates student involvement 12. Positive role model 13. Open-minded 14. Sensitivity 15. Scholarly teaching/knowledge 16. Accessibility 17. Communication skills 18. Individualizes teaching 19. Timely feedback 20. Actively teaches 21. Stimulates effective discussions | The item with the highest rated by both preceptors and students as the highest importance was clinical competence/Judgement followed by positive role model. Students rate stimulates student involvement third, communication skills third. Both students and preceptors rated ego/self-assurance and sensitivity the least important.  All 21 were considered important | Limitations: self-reported. Amount of precepting experience and practice location not addressed |
| 17 | Lewis | 1990 | America: hospital | Medicine | Quantitative study of attendings along with qualitative interviews | Residents (N=24)  Faculty (n=7)  Chief resident (n=3) | **Resident rating of faculty and chief resident preceptors**  16 item form using a visual unnumbered scale. Measurements then taken from 0 to 10 | Quantitative rating form   1. I received the help I asked for 2. I Received concrete instruction about how to proceed 3. Effectiveness of clinical supervision by phone 4. Effectiveness of clinical supervision in person 5. Comfort level with his/her clinical decision making 6. Advocates for the resident 7. Actively involved in patient care 8. Availability 9. Approachability 10. Teaching effectiveness 11. Teaching characteristics (knowledgeable, clear, organised) 12. Teaching skills (constructive feedback, explains well) 13. Adequate coverage of content 14. Professional role model 15. Right amount of autonomy 16. Stimulates autonomy | Most frequently characteristic was “supportive” (17.2%) followed by Supervisory style (15.4%) and then teaching skill (11.1%)  The rankings of preceptors on both qualitative and quantitative measures were similar, but the information revealed by the qualitative method was more specific and used for guiding faculty development. The quantitative measures helped to identify faculty members having difficulty.  Both faculty and students found the interview more beneficial than the rating form.  **Labour intensive.** | Showed interrater reliability.  Both quant and qual methods gave similar rankings of faculty preceptors.  Single site – generalizability?? |
| 18 | Lie | 2009 | America: Community setting | Medicine | Observational study and student evaluation.  Single school – 3 cohorts | Preceptors (N=83) | **Student evaluation of preceptor**  Narrative responses coded for themes.  11 items student evaluation (5 point likert scale 1=strongly disagree 5=strongly agree | **Student Evaluation**  1. My preceptor was well informed about my learning needs  2. My preceptor observed me with at least one patient  3. My preceptor provided opportunities for physical examination  4. My preceptor provided feedback on my clinical skills  5. My preceptor encouraged me to present my ideas  6. My preceptor’s patients seemed comfortable working with a medical student  7. I felt my preceptor took time to teach  8. My preceptor was an excellent role model  9. My preceptor allowed me to interview patients  10. My preceptor used the internet for teaching patient care  11. I was well oriented for my CSE  **Narrative themes emerged**  QUESTION 1 THEMES – GOOD QUALITIES OF PRECEPTOR  Categories and frequencies   - Allows student to see patients independently (37/129 counts*) - Spends time teaching student (27/129) - Gives feedback – corrects and praises (20/129) - Is a role model (15/129) - Listens to the student (12/129) - Spends time observing student with patient (10/129) - Spends time orienting student (5/129) - Practice is welcoming (2/129)   QUESTION 2 THEMES – SUGGESTED WAYS FOR PRECEPTORS TO IMPROVE  Categories and frequencies   - None – no suggestions (22/68 counts) - Spend time orienting student – specify goals (11/68) - Spend time teaching students (9/68) - Assign homework (6/68) - Allow students to administer physical exam independently (5/68) - Use computer for teaching (4/68) - Allow students to interview patients independently (4/68) - See fewer patients (3/68) - Give feedback (2/68) - Observe student (2/68) | Qualities most highly associated with a good preceptor was “allows student to see patient independently”, “Spends time teaching student” and “gives feedback-corrects and praises”.  Student evaluations improved over the course of 3 years.  Site visits with feedback after the visit seemed to improve teaching behaviours, but could also have been increased exposure to students leading to improvement | Strengths: high number of site visits (80%), also 63-87% response to online evaluation. Combination of data sources were used(direct observation, interview, post rotation evaluation) which provided concordance of results.  Hawthorn effect.  Limitations: Some visits were early in rotation, some late. Presence of observer may confound result. |
| 19 | Melaku | 2016 | Ethiopia: University | Pharmacy | Cross-sectional study looking at perceptions of students and preceptors of teaching behaviours | Students (n=126)  Preceptors(n=23) | 52 item questionnaire based on Sonthisombats 47 item instrument. Measurement on a 3 point ordinal scale 1=behaviour not performed, 2=performed but inadequately, 3 behaviour carried out well | **Communication skills**  Connecting all relevant clinical data into a big picture  Explaining the basis for their actions and decision-making in patient management  Presenting information in an organized way  Answering questions clearly and precisely  Speaks loud enough both in class and bedside  **Preceptors clinical practice and bedside manner**  Broad knowledge applied appropriately  Good relationships with patients  Enthusiasm for patient care and teaching  Encouraging students to raise questions  Role model essential attitudes and skills  Emphasize problem solving skills  Giving students opportunity to ask, discuss and exchange opinion  Accessible to students  Preceptors provision of feedback and evaluation  Explain goals and expectations  Expect student to set their own goals  Set criteria for student performance  Evaluate students appropriately  Encourage students to self-evaluate  Give students positive feedback for good work  Invite comment or criticism | Like Sonthisombats study, preceptors rated themselves more highly then students in 11 of 54 items. Particularly encouraging students to self-evaluate, inviting comments on the preceptors own ideas, setting criteria for student performance and grading students on performance and effort.  The differences highlighted the need to preceptor education to overcome deficiencies in their teaching behaviour | Single institution. Small sample size and short history of clinical pharmacy practice in this university.  Self-reported,  subjective |
| 20 | Mintz | 2015 | Canada: university | Medicine | Validation of program tool | Preceptees(N=119) assessing a preceptor | 25 item preceptor assessment rated using 5-point likert scale 1=strongly disagree and 5=strongly agree) along with a 6 item survey on the tools acceptability | **seven domains of the educational process**  (a) learning climate,  (b) control of the teaching session,  (c) communication of goals,  (d) promoting understanding and retention  (e) evaluation,  (f) feedback, and  (g) promoting self-directed learning.  **25 item tool**  1. Listened to learners  2. Encouraged learners to participate actively in the discussion  3. Expressed respect for learners  4. Encouraged learners to bring up problems  5. Called attention to time  6. Avoided digressions  7. Discouraged external interruptions  8. Stated goals clearly and concisely  9. Stated relevance of goals to learners  10. Prioritized goals  11. Repeated goals periodically  12. Presented well-organized material  13. Explained relationships in material  14. Used blackboard or other visual aids  15. Evaluated learners’ knowledge of factual medical information  16. Evaluated learners’ ability to analyze or synthesize knowledge  17. Evaluated learners’ ability to apply medical knowledge to specific patients  18. Evaluated learners’ medical skills as they apply to specific patients  19. Gave negative (corrective) feedback to learners  20. Explained to learners why he/she was correct or incorrect  21. Offered learners suggestions for improvement  22. Gave feedback frequently  23. Explicitly encouraged further learning  24. Motivated learners to learners to learn on their own  25. Encouraged learners to do outside reading | 84% of participants felt the tool gave an accurate impression of their preceptor and 75% would be willing to fill in bi-monthly. This drops to 25% if asked to fill in weekly.  Factor structure was not confirmed by the 25 item tool | Single centre. Participant sample may not be representative.  Tool relevant to internal medicine preceptors – use for other disciplines not examined.  Tool was anonymous and blinded which limits use for closing faculty performance gaps.  24% did not complete the tool. |
| 21 | Litzelman | 1998 | America: University | Medicine | Factor analysis of evaluation instrument and refinement to practical length | Sample of Student evaluations (N=1581) of teachers (n=178) | 58 item likert scale instrument (1= strongly disagree: 5=Strongly agree). Students completed the evalutions 10 days into a 4 week rotation and at the end of the 4 weeks for 4 months | **seven domains of the educational process**  (a) learning climate,  (b) control of the teaching session,  (c) communication of goals,  (d) promoting understanding and retention  (e) evaluation,  (f) feedback, and  (g) promoting self-directed learning. | Construct validation was completed on half of the samples, then tested for repeatability using the second half of the sample. Factor loadings were completed and items with a loading less than 0.40 or complex or ambiguous were eliminated. Items with low-scale correlation were also removed. Both a 6 and 8 factor model did not replicated well, however the 7 factor model did not changed during replication. This left a 25-item instrument containing 7 factors with a alpha co-efficient of 0.97. The 7 domain categories were empirically validated | Strengths – large – enough items for other factors to emerge if they existed. Enough data for findings to be replicated.  Findings were only based on student evaluations, not peer who may have different views on teaching qualities |
| 22 | Skeff | 1988 | America: University | Medicine | Conference presentation |  |  | **7 factors influencing effectiveness of faculty as ambulatory care teachers**   1. Establishing positive learning climate 2. Control of the teaching session 3. Communication of goals 4. Enhancing understanding and retention 5. Evaluation - 6. Feedback 7. Self-directed learning - modelling | Conference presentation |  |
| 23 | Schol | 2001 | Belgium : university | Medicine | Testing validity, reliability, acceptability of a multi-station skills assessment test in a single site | GP, preceptors (N=35) | 2 observers scored 7 standardized simulation activities on a 5 point scale 1=not done or very poor, 5= very good | 7 stage multi station skills assessment:  Station 1 – drawing up a learning agenda  Station 2 – leading an advisory consultation  Station 3 – exchange of information about practice visits  Station 4 – operational case related discussion  Station 5 – feedback conversation  Station 6 – demonstration  Station 7 – an intermediate evaluation conversation | Inter-observer reliability was rated as high to very high on 5 of the seven stations and the separate stations had good internal consistency (Chronbach alpha=0.85) suggesting the multi-station skills assessment is a reliable measure of preceptor teaching skills | Good reliability and consistency but only a single site and no re-test |
| 24 | Sonthisombat | 2008 | Thailand: university | Pharmacy | Study comparing preceptors and preceptees perceptions of the preceptors teaching behaviours | Preceptees (N=76-99% response), Preceptors (N=27-55% response) | 47 item survey of teaching behaviours rating using a 3 point scale of   - Not Done - Done but inadequate - Adequate and well done | Teaching behaviours  Possessing and demonstrating broad knowledge suitable for management of patients in the settings  Applying appropriate updated knowledge to individual patients  Having good relationship with patients Showing enthusiasm in providing patient care Demonstrating sensitivity to patient needs Providing good care to patients Applying updated information from related  fields to individual patients Assigning numbers of patients to take care  of based on student capability Encouraging students to raise questions for  solving patient problems Encouraging students to express their own  feelings and opinions in relation to particular  patients or problems Providing a role model of essential attitudes  and skills in practice Being a good mentor Emphasizing problem solving skills Facilitating student participation in practice Encouraging students to think independently  for resolving problems Using questions to stimulate student learning Helping students in changing and improving  practical skills Capturing learner attentions while teaching Demonstrating enthusiasm for teaching Demonstrating sensitivity and supportiveness  to the students Using questions to stimulate recall of previous  learning and collect them together Closely supervising students to help facilitate  the learning experience Giving student opportunity to ask, discuss  and exchange opinions Spending sufficient time with students Remaining accessible to students when help is needed Discussing practical applications of knowledge and skills | Most students rated their preceptors in the well done range and this study found that preceptors did not overate teaching behaviours of Communication, but did overate 12% of the skills in practice and 25% of the feedback and evaluation.  Preceptors feel they are better at giving feedback, grading students, being open and positive with feedback regarding their teaching.  There was general agreement in 35 of the 47 teaching behaviours | Assessment was subjective. Their may be recall bias as the surveys were a month after the experience. Authors note that the findings may not correspond to other groups of preceptors |
| 25 | Srinivasan | 2011 | America: Hospital | Medicine | Framework development based on Literature review – delphi process, and two additional rounds of consultation | N/a | n/A | **six core competencies**  medical (or content) knowledge, (2) learner-centeredness, (3) interpersonal and communication skills, (4) professionalism and role modelling, (5) practice-based reflection, and (6) systems-based practice. |  |  |
| 26 | Stuart | 1980 | America: Hospital | Medicine | Observational study of preceptor- resident interaction with follow-feedback and re-evaluation 6 months post by both faculty observer blind evaluator who scored all videoed interactions blinded to whether it was initial or post | Preceptors (N=8) | Interactions rated on scale of 0 to 4.  0= category not discussed  4= Category constructively discussed an reviewer felt an appropriate amount of time devoted to that category | Resident-preceptor related categories  -Resident relationship- establish effective relationship, gaining respect, rapport, credibility  - teaching method- vary teaching approach according to awareness as manifested in resident behaviour to handle criticism and information  - problem solving- concentrate on approach to problem rather than immediate solution  - flexible methodology- allow resident experience of different methodologies  - Experience – Draw on preceptors own experience | Both evaluators scored improvement in preceptor performance in all categories after the intervention although the “blind” evaluator gave higher scores. Increases were statistically significant | Small sample number. Variance between raters was measured and was consistent across the sample.  Expensive evaluation method |
| 27 | Walter | 2017 | Canada: University | Pharmacy | Literature review and framework development | N/A | Thematic analysis of 8 relevant articles identifying preceptor competencies and performance indicators | **Proposed Competencies and performance indicators**  **1. Demonstrate a commitment to teaching as a means for growth and skill development for each learner** - • Inspires and motivates learners to develop patient centred services  • Adheres to ethical principles in teaching, demonstrating compassion and integrity • Invests in each learners growth and skill development• Teaches by example• Designs placement activities to meet course goals and objectives • Engages in activities to continually develop teaching skills  **2. Create practice-based learning opportunities by promoting active collaboration in client care -** • Aware of placement goals and objectives and plans activities to meet them• Promotes student participation at rounds, team meetings, and conferences. • Promotes collaboration and teamwork between student pharmacist, team members and patients • Provides learners with graduated responsibility based on their abilities • Provides learners the opportunity to learn with, from and about the roles and responsibilities of other health professionals  **3. Engage in continuous reflection, self-assessment and lifelong learning to improve their effectiveness as educators (Continuing Professional Development)** - • Seeks feedback to identify strengths and limitations in teaching competence• Reflect on one's own teaching and practice routinely • Evaluate the outcomes of their teaching• Develops personal educational goals based on reflection and self-assessment and implements a plan to achieve those goals  **4. Demonstrate effective communication skills** - • Communicates expectations, goals and information in ways that stimulate and engage  learners• Exhibits excellent interpersonal skills in interprofessional teams• Promotes continuity of student support and  supervision through communication • Constructively addresses conflicts or disagreements  **5. Create professional relationships with students** - • Demonstrates respect for each learner • Demonstrates a caring attitude towards student pharmacists (i.e. gets to know the student goals) • Accessible to student pharmacists• Open to alternative approaches to solve problems and issues • Creates an environment that facilitates learning  **6. Adapt to students’ learning needs -** • Considers how individual students learn (i.e. learning styles) • Determines each learner's prior knowledge and skills (strengths and barriers) • Utilizes educational techniques that are appropriate for the student and/or content • Provide sufficient practice opportunities to address both course objectives and student's learning needs  **7. Model best educational and clinical practices to facilitate** development of skills - • Keeps up-to-date on educational practices and resources within their field of expertise • Provide adequate demonstration and coaching of skills• Break complex concepts and skills into separate steps• Models professional practice standards in their field  And Demonstrates effective collaboration within practice environment  **8. Facilitate student development of critical thinking, problem solving and decision making skills -** • Use open-ended questions to promote learning - Encourage student to question practice and evaluate care provided • Engage in reflection with student to link theory to practice • Problem solve in a social context • Explore the student's assumptions, values, feelings, and perspectives that influence understanding of experiences and actions  **9. Assess and document student pharmacist performance –**  • Assess learners progress in acquiring knowledge skills and attitudes  • Gives specific, timely and balanced feedback that identifies strengths and areas of improvement• Adjust supervision and responsibility based on student's abilities • Document student progress and performance  assessments in placement records | Recurring Competencies in the pharmacy literature included:  *commitment to teaching, role modelling, and encouraging*  *self-directed learning.*  Across all disciplines, the following were consistent :  *the ability to develop a relationship with the student, demonstrate skill and adaptability in*  *teaching, and the ability to apply the principles of lifelong learning were consistently required competencies*  Preceptors must:  *demonstrate knowledge and skill in their clinical area or field*  Ability to provide effective feedback was the only addition to the PEP-C qualities previously developed | Strengths of the study: Included international, multidisciplinary literature and an iterative process that included Canadian and America faculty with experiential education expertise.  No Delphi process to get a better consensus on the competencies or performance indicators |
| 28 | Conigliaro | 2010 | America: university | Medicine | Literature review informing development and validation of 15 item OSCE type checklist | Clinical teachers (N=9) | OSCE checklist with 3 point scale of 1=never  2=sometimes  3=always  Rated by 2 randomly assigned observers on 2 occasions | Checklist items  1 Clerkship goals and expectations were reviewed  2 Rounds were begun and ended on time  3 Literature searches or topic discussions were assigned  4 Students’ history and physicals were reviewed  5 Students were treated as team members  6 A broad knowledge of clinical issues was demonstrated  7 Education and patient management were balanced  8 Students were encouraged to formulate their own assessment and plan  9 Students were encouraged to ask questions  10 Constructive feedback was provided  11 Physical findings were reviewed and demonstrated  12 Clinical decisions (e.g. selection ⁄ interpretation of tests, etc.) were explained  13 Clinical findings (e.g. X-rays, CT scans, etc.) were reviewed and demonstrated  14 Professional and ethical behaviours were modelled  15 Effective interpersonal and communication skills were modelled | Internal consistency of items was found to be good with e, but there was 64% variation between the persons rating. Raters assessed similarly on the 2 occasions, but there was variation between raters. It was calculated that 3 raters would be needed to ensure generalisability was acceptable.  It is suggested that this method may be of use when student evaluations identify poor performance of clinical teachers | Single institution and small sample number with few variations. There is a possibility that there can be changes to clinical teaching behaviour between the times of the 2 observations which could confound |
| 29 | Harris | 2012 | America: college | Pharmacy | Committee meeting Report on necessary features of preceptor development and recognition of excellence  Based on literature search | N/A | Identifying competencies and indicators of performance from literature review | 6 Pharmacy Preceptor Competencies  **Possess Leadership and Management Skills**   - Demonstrates effective managerial and leadership relationships with colleagues and student pharmacists - Demonstrates humility and is self-reflective related to own limitations - Monitors quality of own professional practice, practice at the site, and teaching activities - Demonstrates non-discriminatory behaviour - Embodies Practice Philosophy - Motivates and inspires pharmacists and student pharmacists to develop patient-centred care services - Has a mission and/or vision for pharmacy illustrated by a strategic plan that encourages planning, implementation, and evaluation of programs, initiatives, and standards at their practice site - Insists that administration or ownership supports pharmaceutical care services   Role Model Practitioner   - Provides or supports high-quality patient-centred care or pharmacy-related services - Practices ethically - Practice patterned after accepted guidelines or model practices - Solves problems effectively using sound critical thinking and problem-solving skills - Educates and integrates patients, family members, and caregivers in decision making - Interacts professionally with patients, health care providers, and team members   Commitment to excellence in scholarly teaching   - Demonstrates a caring attitude toward student pharmacists (e.g., gets to know them, inquiries about previous experiences and future career goals) - Provides prompt assessment and constructive feedback - Promotes cooperation between student pharmacists and other members of the health care team (e.g., physicians, nurses, social workers, lab technicians, etc.) - Is accessible to student pharmacists - Aids student pharmacist learning beyond the normal/regular practice experience - Teaches by example (e.g., demonstrates patient assessment skills, discusses the clinical reasoning process, practices ethically) - Inspires and motivates student pharmacists - Engages in activities to continually develop teaching skills - Invites and accepts constructive feedback to improve teaching skills Incorporates new techniques to promote learning   Effective communication skills   - Demonstrates enthusiasm and passion for his or her practice - Displays compassion and is caring toward patients - Commands student pharmacist attention and maintains it - Provides substantive information and clarifies when necessary Raises thought-provoking and significant questions instead of just providing answers - Demonstrates awareness and understanding of how cultural elements (e.g., beliefs, values, practices) can impact patient behaviours, health communication and patient outcomes - Exhibits excellent interpersonal skills within an interprofessional healthcare team and/or environment   Encourages self-directed learning   - Assesses the student pharmacist’s baseline knowledge, skills, and abilities - Defines objectives and expectations for the practice experience - Develops plan to meet objectives for practice experience (activities, experiences, assignments, reading, reflections) - Teaches to the level of the student pharmacist - Models appropriate skills and behaviour for practice setting   (e.g., efficiency)   - Coaches student pharmacist behaviour through effective, constructive, and timely feedback - Creates a safe yet challenging learning environment - Treats student pharmacists as colleagues-in-training - Encourages student pharmacists to be independent, lifelong learners - Respects student pharmacist’s individuality with fairness and balance - Recognizes and teaches to different learning styles Integrates and includes student pharmacists into practice experience - Stimulates and engages the student pharmacist in critical thinking skills - Encourages student pharmacist’s self-reflection on quality of tasks and efficiency of tasks | Concept of “Master Preceptor” – Preceptors should be assessed on these 6 competencies and measured against the indicators of performance as well as being reassessed every 1-2 years after initial training development.  Assessment discussed as student evaluation, site visit, preceptor self-assessment, completion of preceptor development modules.  The master preceptor should not just satisfy the competencies but demonstrate them over time.  Recognition should be by industry bodies overseen by the peak body to celebrate and reward excellence |  |
| 30 | Griffith | 2000 | America: University | Medicine | Cohort study | Students (N=484)  Preceptors (N=46) | Scores on medical board examinations vs high rated attendings or low rated attendings | Students examination outcomes | Students who worked with at least One high rated preceptor scored higher on post clerkship NBME exam and USMLE step 2 exam | Single institution, single discipline and one outcome measure |
| 31 | AL-Arifi | 2018 | Saudi | Pharmacy | Cross-sectional study | N=52 students | Questionnaire 4 point scale  Excellent, very good, satisfactory and needs improvement  Also  Not satisfied  Somewhat satisfied  Satisfied  Very satisfied | Attributes assessed by students  -Welcoming  - identified previous knowledge and skills and set goals.  -Knowledge of clinical, environmental and patient needs  -approachable and clear communication  - assisted in supporting learning  -Role modelled professional practice and patient centre care  -Created learning opportunities and provided safe learning environment  -Offered regular specific and constructive feedback  -Likes to teach | Overall Students were somewhat satisfied with preceptors teaching behaviour in communication, practice, feedback and evaluation | Single site  Small cohort  Previously validated questionnaire used |
| 32 | Bochenek | 2016 | USA/hospital | Pharmacy | Quantitative survey instrument | N=69 | 55 item web-based survey of preceptee perception of preceptors as role models and areas of improvement: 5 point likert scale strongly agree to strongly disagree | Qualities of role models:   1. Clinical – empathy, communication, enthusiasm, clinical reasoning skill, professional competence 2. Interest in teaching, establishes rapport, available, positive attitude, feedback, understands learners needs, encourages active participation, Well organised, aware of status as role model. 3. Personal Qualities – patients, honesty and integrity, easy to work with, self-confidence, leadership qualities, involved in professional organisations. | Preceptees rated as desirable all qualities highly except involvement in professional organisations, and rated current preceptors lowest on being organised, providing feedback. | Residents with multiple preceptors rated only 1. Single site. Survey done early in residency. Original survey validated, but adaptions not validated |
| 33 | Borimnejad | 2018 | Iran/Hospital | Nursing | Qualitative interviews | n=6 | Interviews with preceptors indentifying attributes of preceptors | Patience, empathy, self-sacrifice, non-judgemental | Empathy of the most significant characteristics, then being non-judgemental and patient | Small sample, single institution |
| 34 | Brink | 2018 | USA | Medicine | Delphi | n=40 participants | 2 delphi rounds identifying teaching competencies of medical educators | 21 competencies in 5 domains identified.  Learner centeredness  Demonstrate a commitment to the learners’ success and well-being leading  to the learners’ growth in to their professional roles.  1. Prepare the clinical environment, including staff, patients, and other colleagues, for the learner.  2. Orient the learner to the community, to local resources, and to the clinical environment.  3. Ascertain each learner’s knowledge, skills and attitudes related to rotation expectations and link to your patient’s/clinic population.  4. Assess and respond to the learner’s cultural context.  5. Help learners develop learning goals aligned with patients’ needs.  Interpersonal and communication skills  Teach and communicate effectively.  1. Clearly communicate expectations to the learner.  2. Tailor precepting style to the needs of the learner.  3. Identify barriers to learning (eg. housing, geography, psychological, economic,  family, etc).  4. Maintain a safe learning environment for the student (i.e., approachable,  supportive, encouraging, student can admit limitations).  Professionalism and role modeling  Demonstrate  best educational and evidence- based practices and role model those behaviors for learners.  1. Display enthusiasm for teaching.  2. Respectfully respond to each learner’s unique needs and learning goals related to patient care.  3. Be available and accessible to learners.  4. Acknowledge when beliefs/attitudes are influencing the teaching/learning  environment.  5. Model highest standards of the profession.  Practice-based reflection and improvement  Role model continuous self- assessment and lifelong learning.  1. Model the appropriate use of evidence-based medicine in clinical practice. 2. Seek feedback from the learner and rotation director; identify and act on  improvement goals.  3. Engage in continuous learning as physician and teacher with targeted  teaching goals.  Learner assessment  Provide appropriate feedback.  1. Solicit student self-assessment.  2. Provide timely formative and actionable feedback to the learner regarding  their progress (eg, learning goals; rotation competencies; knowledge, skills,  and attitudes).  3. Check that formative feedback was heard and understood and that the  learner initiated a feasible action plan.  4. Provide summative feedback to the learner and the medical institution. | 21 competencies in 5 domains identified. | Broad range of participants supports validity when using Delphi method  40 participants.  Skewed towards medical profession |
| 35 | Ferreira | 2017 | Brazil, | Nursing | Qualitative, descriptive study | n=6 nurse preceptors | Qualitative interviews | 1. Continuing education 2. Planning according to student need 3. Demonstrate knowledge into action | Descriptive paper of nurses perceptions of what is needed to be a preceptor | Small sample in a single institution |
| 36 | Heshmati-Nebavi | 2009 | Iran | Nursing | Qualitative- grounded theory | n=4 students  n=6 nursing educated | Interviews on effective clinical teaching experiences | 1. Empathy 2. Affection for the profession 3. Reflective thinking 4. Clinical knowledge 5. Curriculum knowledge 6. Making learning enjoyable 7. Turning theory into practice 8. Clinical reasoning 9. Patient centred 10. Forming positive relationships with students 11. Working as part of a team 12. Being a role model | 12 traits identified as being important competencies of being a preceptor | Study relevant to one context – Nursing in Iran.  Small sample |
| 37 | Jahangiri | 2012 | USA | Dentistry | Qualitative | N=157 | Qualities most liked and least liked in clinical teachers in dentistry as asked in a written survey | Character, Competence and Communication  Character: caring, motivation, empathy, patience, professionalism, available, fairness,  happiness, patient-centred  Competence: knowledgeable, expertise, efficient, skilful, effective  Communication: feedback,  approachable and interpersonal communication | 17 Categories across the three themes were identified | Consistent with other studies |
| 38 | L’Ecuyer | 2018 | USA | Nursing | Descriptive qualitative study | N=553 preceptor course participants | Qualitative survey responses defining preceptor competency after attending a 1-day course | Knowledge: expertise, learning styles, personality, role preparation, emotional intelligence  Skill: Communication, flexibility, feedback, interpersonal skills, organisation, role model, open to improving skill, critical thinking, goal setting, protector.  Attitude: patience, desire to precept, understanding, approachable, kind, confident, trustworthy, positive attitude | Survey response coded and assigned to three categorises | Demographics of participants not identified, questions were open to some interpretation, but a large sample, but one discipline (nursing ) and further validity testing needed. |
| 39 | Lee | 2002 | Australia | Nursing | Quantitative | n=121 | Use of the Nursing Clinical teacher effectiveness instrument – 47 item checklist which rates characteristics of effective clinical teachers – 5 point likert scale | - Good role model - Confident - Encourages climate of mutual respect - Demonstrates clinical judgement - Demonstrates clinical skills - Supports and encourages students - Listens attentively - Identifies and uses practice opportunities - Communication skills - Appropriate feedback skills - Questions students to elicit reasoning - Gears learning to students’ level - Open minded - Non-judgemental | Authors concluded that the ability to adapt teaching to students knowledge and experience is paramount. Also a greater emphasis on teaching ability | Small sample size,  One cohort was excluded due to low response rate. |
| 40 | Stenfors-Hayes | 2010 | Sweden | medicine | Qualitative- semi-structured interviews | N=39 | Analysis of semi-structured interviews – question, what does it mean to be a good teacher, what does it mean to be a good supervisor and what differences in these roles | A good teacher focuses on students’ learning  A good teacher responds to students’ content requests  A good teacher conveys knowledge  A good clinical supervisor stimulates students’ growth  A good clinical supervisor shares what it is like to be a doctor  A good clinical supervisor shows how things are done | Study examined differences in the perception of the role of teacher and supervisor in clinical setting – teaching seen as less individual while supervision more one on one, show, then do. | Single institution  Only one professional field studied.  Not all participants had the same teacher or supervision training which could influence their perception of the roles |
| 41 | Sutkin | 2008 | USA | Medicine | Literature review | N=68 articles | Literature review | - Medical/clinical knowledge - Clinical/technical skill -competence - Create positive learning environment and relationship with students - Communication skills - Enthusiasm for medicine/teaching/general | 2/3 of descriptions and themes identified as non-cognitive  -“Whereas cognitive abilities generally involve skills that may be taught and learned, albeit with difficulty, noncognitive abilities represent personal attributes, such as relationship skills, personality types, and emotional states, which are more difficult to develop and teach. “- Sutkin | 2 reviewers independently identified articles followed a rigorous systematic approach |
