# Supplementary material for: Measuring and assessing the competencies of preceptors in health professions: a systematic scoping review
Source: BMC Med Educ. 2020 May 24;20:165. doi: 10.1186/s12909-020-02082-9 (PMC7247189; doi:10.1186/s12909-020-02082-9)
Supplement: Supplementary file 3 — Additional file 3. Appendix 3 – GRADE CERQual evaluation table. [file 12909_2020_2082_MOESM3_ESM.docx]

|  | (1-41) |  |  |  |  |  |  | |
| --- | --- | --- | --- | --- | --- | --- | --- | --- |
| **Mode of assessments** – Methods used to measure and assess the competency of preceptors include student evaluation, peer evaluation, self-evaluation and students exam performance. The validity of the method, whether it be a survey instrument or an observational study, qualitative interviews needs to be considered along with the acceptability of the method. | **Student evaluation of preceptor**  (1, 3, 6, 8, 9, 16, 17, 19, 20, 22, 26, 30, 38) | **Moderate methodological limitations**  -Small sample(3, 16, 19, 20, 22, 26, 30, 38)  -Observer effect (Hawthorne)-(3, 19, 20)  -Reliability/Validity not established -(3, 8, 9, 17, 22) moderate concerns  Subjective measure- (3, 16, 19, 20, 22, 26)  Process issues (1, 6) -moderate concerns | **No or Minor concerns**: Descriptive finding that is well supported by the data | **Moderate concerns:**  Data Quantity: 21 of the studies were considered small sample size, from a single cohort or site.  Richness: little regard was given to the heterogeneity of the data. | **No or Minor concerns:**  3 studies made mention of the possibility that their results may not be applicable to other settings, however this was assessed as minor due to this being an untested concern and leaves open the possibility of testing in further study.  Context, phenomenon of interest and perspective relevant. | **Moderate-High Confidence** | 13 studies with moderate methodological limitations: small sample size, subjectivity and possibility of observer effect. Moderate concerns about adequacy of the data and Minor concerns about coherence and relevance | |
|  | **Peer evaluation** (3, 4, 7, 10, 13, 14, 24, 28) | **Moderate methodological limitations**  -Small sample (3, 4, 6, 7, 10, 13, 14, 24, 28)  Reliability/Validity not established(3, 6, 10, 13)  - Observer effect -(3, 4, 7, 13, 14, 24, 28)  - Subjective measure (3, 4, 6, 7, 10, 13, 14, 24, 28) | **No or Minor concerns**: Descriptive finding that is well supported by the data  **No or Minor concerns**: Descriptive finding that is well supported by the data | **Moderate concerns**  Small samples give some concern about quantity. Some papers descriptive without results. Inclusion of qualitative papers with detailed observations adds to richness of the data  **Moderate concerns**  Small samples and descriptive papers lead to concerns about quantity and richness of the data | **No Concerns**  The setting, perspective, phenomenon of interest, context is relevant to the finding  **Minor Concerns**  perspective, phenomenon of interest, context is relevant to the finding  2 studies have concerns about relevance outside the study setting | **Moderate confidence**  **Moderate confidence** | Moderate concern about methodology and adequacy of data |  |
|  | **Self-evaluation** (1, 9, 13, 15, 22, 26) | **Moderate methodological limitations**  - Small sample (13, 15, 22, 26)  - Reliability/Validity not established -(1, 9, 13, 22, 26)-  Process issues(1, 13, 15) |  |  |  |  | Moderate concern about methodology and adequacy of data |  |
|  | **Students Exam Performance(41)** | **Minor concerns**  -Subjective measure of preceptor quality rating(41)  - Single site | **Minor concerns**: Descriptive finding that is supported by the data although data is limited. | **Major concerns**  This is the only paper that describes this measure leading to concern with quantity and richness of data | **Moderate concerns**  Study in one setting and on leads to concern about relevance to other contexts | **Low confidence** | Moderate concern with relevance and major concern regarding adequacy | |
|  | **Method Validity** (1-6, 13, 21-24, 26) | **Moderate methodological limitations**  -Small Sample (1-4, 6, 21-23, 25, 26)  - Reliability/Validity not established (1, 3, 13, 22, 23)  -Process issues(1, 2, 5, 6, 20, 26) included incorrect links sent, recruitment process issues, very small sample, recall bias – moderate concerns  -Hawthorne effect-(1, 3, 4, 6, 21, 22, 24, 26)  - Subjective measure(1, 3, 4, 6, 13, 22, 24, 26) |  |  |  |  |  |  |
| **Student evaluation of preceptor is a valid method of assessment when using an appropriate tool and can help identify preceptors who may require training assistance.** | (2, 3, 6, 16, 17, 19-21, 23, 25) | **Minor methodological concerns:**  -Low response rate(2, 3)  -Hawthorne effect(2, 3, 16, 19)  - single site(16, 19, 21)  -subjective(2, 3, 16, 19) | **No or minor concerns:**  The finding is well supported by the data | **No or Minor concerns:**  10 studies contributed to the finding and is supported by both quantitative and qualitative data, although some data is based on small samples. | **Minor concerns**  The setting, perspective, phenomenon of interest, context is relevant to the finding | **HIGH confidence** |  | |
| **Preceptors can overestimate their abilities in a self-evaluation when compared to student evaluations** | (1, 22, 26) | **Moderate methodological concerns:**  Sample size(1, 22, 26)  -process issues(1)  -Not validated(1, 22, 26)  -Subjective(1, 22, 26)  -Hawthorne effect(1, 22, 26)  - recall bias(26) | **Moderate concerns:**  2 studies(22, 26) were similar, based on the same instrument, came to the same overall conclusion, but had some variation in the areas of difference. | **Serious concerns:**  All small samples sizes and single site, single cohort studies  One study was a new pharmacy school with limited teaching experience in pharmacy practice(22) | **Moderate concerns:**  results may not be applicable to other settings; however this was assessed as moderate due to this being an untested concern and leaves open the possibility of testing in further study. | **Low confidence** | Moderate methodological, relevance amd coherence concerns, serious concerns with adequacy | |
| **Qualitative evaluations have been found to provide more specificity and detail.** | **(6, 16, 19, 20)** | **Minor concerns**  -Single school(16, 19, 20)  -Small sample (19)  -Observations did not occur at the same point for all participants(20)  -Hawthorne effect(20)  -unable to match student responses to specific sites(20) | **No or Minor concerns:**  The finding is well supported by the data | **Minor Concerns:**  Small sample size (19)and results from single schools (16, 19, 20) do not significantly impact the adequacy of the data as the | **Minor Concerns:**  The strongest contributors to the finding are all from Medicine in the USA but does not impact the review finding | **High confidence** | Minor concerns regarding methodology, relevance and adequacy do not impact the finding | |
| **Qualitative evaluations are more difficult to institute, more resource intensive and not as acceptable in large scale.** | **(6, 16, 19, 20, 24)** | **Minor concerns**  -Single site(16, 19, 20, 24)  -Small sample(19, 24)  -Hawthorne effect(20, 24) | **No or Minor concerns:**  The finding is well supported by the data | **Moderate concerns:**  2 contributing studies had small sample sizes, and all were from single sites, but the study method provided a rich source of information | **No or Minor concerns:**  The finding is supported by the data. Consistent across a range of settings including medicine, nursing and pharmacy | **Moderate Confidence** | Minor concerns regarding methodology and moderate concerns regarding adequacy | |
| **Assessment of preceptors can inform training needs as part of preceptor development programs** | **(1, 6, 9, 11, 16, 18-20, 22, 26)** | **Minor concerns**  -Informed by research, not evaluated (9, 11)  -Single site(16, 22, 26)  -Self-reported(18)  -Differences in experience not addressed(18)  -geographical differences may influence results(18, 22, 26)  -Hawthorne effect(20, 22, 26)  -Student evaluations not linked to specific preceptors(20)  - indicators synthesised from literature review and consulting clinical education experts-details not outlined(11) | **No or minor concerns:**  The finding is well supported by the data | **No or minor concerns:**  Despite 3 Studies discussing geographic situation and 3 studies having singles sites, the body of evidence spans a range of geographic locations and sites providing adequacy of data.  3 studies were qualitative, providing rich data sources from adequate sample sizes | **No or minor concerns:**  The finding is relevant and consistent across a range of settings including medicine, nursing and pharmacy | **High confidence** | Minor concerns with the methodology of studies but not impacting significance of the finding. | |
| **Assessment of preceptors can serve as the basis for selection of candidates for preceptor roles** | **(7, 10, 11, 13)** | **Major concerns**  Descriptive papers no results reported. (10, 11, 13)  No intervention(10, 11, 13)  indicators synthesised from literature review and consulting clinical education experts-details not outlined(11) | **Minor concerns:**  Descriptive finding supported by 2 descriptive papers and one paper based on literature review and expert opinion | **Serious concerns:**  3 papers offering thin data based on descriptive papers with no evaluation leading to concern about quantity and richness of the data in this respect | **Moderate concerns:**  The setting, perspective, phenomenon of interest, context of all papers is relevant, but there are no results described | **Low confidence** | Serious concerns regarding adequacy, major concerns regarding methodology and moderate concerns regarding relevancy | |
| **Assessment of preceptors can serve as the basis for career progression and recognition** | **(7, 10, 11, 13)** | **Major concerns**  Descriptive papers. No evaluation(10, 11, 13)  No intervention(10, 11, 13)  Indicators synthesised from literature review and consulting clinical education experts-details not outlined(11) | **Minor concerns:**  Descriptive finding supported by 2 descriptive papers and one paper based on literature review and expert opinion | **Serious concerns:**  3 papers offering thin data based on descriptive papers with no evaluation leading to concern about quantity and richness of the data in this respect | **Moderate concerns:**  The setting, perspective, phenomenon of interest, context of all papers is relevant, but there are no results described | **Low confidence** | Serious concerns regarding adequacy, major concerns regarding methodology and moderate concerns regarding relevancy | |
| **Identified competencies with associated measures of performance** | **-Leadership and management skills (12, 31)** | **Minor concerns:**  Committee report based on literature and expert opinion informing policy of American association of colleges of pharmacy,  No intervention.(12)  Single site(31)  Survey early in residence(31) | **Minor concerns:**  Two sources identify this finding. | **Serious concerns:**  2 papers contributing, not a rich source of data | **Moderate concerns:**  Setting and context is Pharmacy in USA. Phenomenon of interest is relevant to the finding | **Low to moderate confidence** | Serious concerns regarding adequacy of data.  Moderate concerns regarding coherence and relevance. | |
|  | **-Role model practitioner(3, 5, 6, 11, 12, 14-17, 27, 28, 30, 31, 33, 35, 37, 38)** | **Moderate concerns:**  Small sample(3, 5, 6, 14, 30, 35, 38)  Hawthorne effect(3, 14)  Length of time to complete a barrier(3, 5)  Low response rate(5, 38)  Uncertainly over reaching saturation(5)  Observers not trained for consistency(6)  Instrument not validated(6, 11)  No results reported(11)  Single site (16, 30, 31)  No results reported(11)  Based on consensus(12, 27, 29) | **No or minor concerns:**  Descriptive finding that is well supported by the data | **No or minor concerns:**  6 studies with small sample sizes leads to minor concern regarding quantity of data. A mix of validated qualitative studies and quantitative studies provide rich source of data, but thin in these small studies  A large number of studies contributing to the finding | **No or minor concerns:**  The setting, perspective, phenomenon of interest, context is relevant to the finding | **Moderate to high confidence** | Moderate methodological concerns and concerns regarding quantity of data | |
|  | **-Commitment to excellence in teaching (3, 5, 11, 12, 14-16, 29, 33)** | **Moderate Concerns**  Small sample(3, 5, 14)  Hawthorne effect(3, 14)  Length of time to complete a barrier(3, 5)  Low response rate(5)  Uncertainly over reaching saturation(5)  Single site (16)  No results reported(11)  Based on consensus(12, 27, 29)  Not validated(6, 12, 27, 29) | **No or minor concerns**  Descriptive finding that is well supported by the data. 3 studies based on consensus lend weight to coherence | **Minor concerns:**  3 studies with small sample sizes leads to concern regarding quantity of data. A mix of validated qualitative studies and quantitative studies provide rich source of data, Dephi with a broad range of participants adds to richness if data | **No or minor concerns:**  The setting, perspective, phenomenon of interest, context is relevant to the finding | **Moderate to high confidence** | Moderate methodological concerns and concerns regarding quantity of data | |
|  | **-Adapts to the learning needs of students (3, 16, 18, 27-29, 34)** | **Moderate Concerns**  Small sample (3, 34)  Single site (3, 16, 34)  Based on consensus (27, 29)  Not validated(27, 29) | **No or minor concerns**  Descriptive finding that is well supported by the data. | **Minor concerns:**  Single site and small samples lead to minor concerns about quantity and richness of the data. Descriptive qualitative study added a rich source | **No or minor concerns:**  The settings, perspective, phenomenon of interest, context is relevant to the finding | **Moderate to high confidence** | Moderate methodological concerns and minor concerns regarding adequacy of data | |
|  | **-Demonstrates respect for the learner (14, 17, 18, 28)** | **Moderate Concerns**  Small sample(14, 28)  Single site(14, 17, 28)  Tool designed for different setting(18) | **No or minor concerns**  Descriptive finding that is well supported by the data. | **Moderate concerns:**  Single site and small samples lead to concerns about quantity and richness of the data | **Moderate concerns:**  Tool used in one paper designed for a different setting and untested in the studied environment.  Context, setting and phenomenon of interest of remaining papers relevant | **Moderate confidence** | Moderate methodological and relevancy concerns and moderate concerns regarding adequacy of data | |
|  | **- Encourage self-directed learning (12, 21, 25, 33)** | **Moderate concerns:**  Single site and single ward(21)  Based on Literature and opinion(12, 25)  Skewed toward medical profession(33) | **moderate concerns**  2 papers(12, 25) talk of importance of but (21) suggests student can’t distinguish this from teacher knowledge | **Moderate concerns:**  2 papers give a range of expert opinion and literature review but not extensive or diverse. One paper has comprehensive validation process | **moderate concerns:**  Context and setting are limited to pharmacy and medicine and America. Phenomenon of interest is relevant. | **Moderate confidence** | Moderate concerns about methodology, relevance, adequacy and coherence | |
|  | **Demonstrate reflective practice (5, 27, 29, 33)** | **Moderate concerns:**  Large number of criteria leading to difficulty in respondents discriminating between them(5)  Low response rate to Delphi(5)  Delphi approach but results not tested(27)  Limited number of expert opinion and guidelines based on literature review(29) | **No or minor concerns:**  3 papers with Delphi approaches and 1 with range of expert opinion supports the descriptive finding | **Moderate concerns:**  One study had low Delphi response rate. One study came to consensus with a Delphi approach, but results have not been tested, another had a limited range of expert opinion and literature review  leading to concerns about quantity and richness of data, | **No or minor concerns:**  The settings, perspective, phenomenon of interest, context is relevant to the finding | **Moderate confidence** | Moderate concerns about methodology and adequacy not impacting the finding | |
|  | **-Effective communication skills**  **(3-6, 11, 12, 14, 18, 22, 26-31, 33, 36-38, 40)** | **Moderate Concerns:**  Small sample size(3, 6, 22, 26, 34, 38)  Small number of tools completed(3)  Single site(14, 22, 26, 34, 38)  Large number of criteria leading to difficulty in respondents discriminating between them(5)  Low response rate to Delphi(5)  Observers not trained for consistency(6)  Based on literature and expert opinion(12, 27, 29)  Study based on characteristics drawn from different setting(18)  Self-reported(18, 22, 26)  Hawthorne effect(3, 6, 14, 22, 26) | **No or minor concerns:**  Descriptive finding that is well supported by the data. | **Minor concerns**  Qualitative and Quantitative studies along with expert opinion and Delphi methods provide good quantity and richness of data despite 4 studies with small samples sizes and one study with low response rate to its Delphi | **Minor concerns:**  The tool used in one study was designed for a different setting, however,  the settings, perspective, phenomenon of interest, context of remaining studies is relevant to the finding | **Moderate to High Confidence** | Moderate concerns regarding methodology, minor concerns for adequacy and relevance do not impact the finding | |
|  | **-Effective provision of feedback(4, 5, 12, 27, 29, 33, 36-38)** | **Moderate concerns**  Small sample(4, 38)  Hawthorne effect(4, 38)  Large number of criteria leading to difficulty in respondents discriminating between them(5)  Low response rate to Delphi(5)  Delphi approach but results not tested(27)  Limited number of expert opinion and guidelines based on literature review(29)  Based on limited consensus(12, 27, 29) | **No or minor concerns:**  3 papers with Delphi approaches and 2 with range of expert opinion supports the descriptive finding | **minor concerns:**  One paper reached consensus with limited number of participants, one Delphi approach to reaching consensus had low response rate and one Delphi had a broad range of participants and good response rate Minor concerns regarding Quantity and richness of data | **No or minor concerns:**  The phenomenon of interest, context and setting of the papers is relevant to the finding | **Moderate to high confidence** | Moderate concerns regarding methodology and adequacy | |
|  | **-Demonstrate patient centred practice**  **(11, 12, 29, 35, 36)** | **Moderate concerns:**  3(11, 12, 29) studies based on a consensus approach based on literature and limited expert opinion  Small sample(34, 35)  Descriptive paper no results(34) | **No or minor concerns:**  Descriptive finding that is well supported by papers contributing to the finding | **Major concerns**  One study (11) did not report results of their study and the make-up of the panel supplying expert opinion was not specified, Another paper(34) was descriptive only. The synthesis of competencies from the literature review were given but no details of the actual review. The remaining two papers are based on consensus and committee reports that are limited. There is a concern that the data may be lacking in quantity and richness. | **Moderate concerns:**  Phenomenon of interest is relevant.  One paper was only relevant to Nursing in Iran. | **Moderate confidence** | Moderate concerns regarding methodology, and relevance. Major concerns regarding adequacy | |
|  | **-Facilitate critical thinking, problem solving and decision-making development**  **(3, 5, 29, 35)** | **Moderate concerns**  Small sample (3)  Single site(3)  Hawthorne effect(3)  Large number of criteria leading to difficulty in respondents discriminating between them(5)  Low response rate to Delphi(5)  Limited number of expert opinion and guidelines based on literature review(29) | **No or minor concerns:**  Descriptive finding that is well supported by papers contributing to the finding | **Moderate concerns**  One study had a small sample, low completion rate of tool and low dephi response. lack of Delphi in another paper. Looking at participants in 2 papers (5, 29) together gives a range covering USA, Canada, UK, Europe and a reasonable degree of richness of data but low quantity. | **Moderate concerns**  Context and setting are pharmacy, medicine and nursing but nursing study only relevant to Iran. Phenomenon of interest is relevant | **Moderate confidence** | Moderate concerns about methodology, adequacy and relevance | |
| **Skills of effective preceptors without indicators of performance.** | **-Organised and ability to prioritize**  **(5, 6, 14, 15)** | **Moderate concerns**  Small sample(6, 14)  Single site(6, 14, 15)  Large number of criteria leading to difficulty in respondents discriminating between them(5)  Low response rate to Delphi(5)  Self-reported (15) | **No or minor concerns:**  Descriptive finding that is well supported by papers contributing to the finding | **Moderate concerns**  3 papers had small samples and single sites and concerns about generalisability outside of the setting. Another paper had low Dephi response rate. 2 Qualitative studies provide rich descriptions, but quantity of data is thin | **Minor concerns**  Settings relevant covering Nursing, Medicine, pharmacy. Phenomenon of interest relevant Concerns about generalisability outside the context studied but does not significantly impact the finding. | **Moderate confidence** | Moderate methodological and adequacy concerns | |
|  | **-Empathetic**  **(3, 5, 18, 32, 35)** | **Moderate concerns:**  Small sample (3, 32, 35)  Single site(3)  Hawthorne effect(3)  Large number of criteria leading to difficulty in respondents discriminating between them(5)  Low response rate to Delphi(5)  Study based on characteristics drawn from different setting(18) | **No or minor concerns:**  Descriptive finding that is well supported by papers contributing to the finding | **Moderate concerns:**  Broad range of Dephi participants in one paper and large sample of preceptors and preceptees in another is countered by the low Dephi response rate and another 2 papers with a small single site sample, leading to moderate concerns about quantity and richness of the data | **Modereate concerns:**  Settings and phenomenon of interest are relevant. Context is the US, Iran and UK. One study used a tool validated in a different setting. Study in Nursing was relevant only to Iran | **Moderate confidence** | Moderate methodological, relevance and adequacy concerns | |
|  | **-Ethical**  **(5, 14, 31)** | **Moderate concerns:**  Small sample(14)  Single site(14, 31)  Large number of criteria leading to difficulty in respondents discriminating between them(5)  Low response rate to Delphi(5) | **No or minor concerns:**  Descriptive finding that is well supported by papers contributing to the finding | **Moderate concerns:**  One paper had a broad range of Delphi participants but mainly UK and low response.  Taiwan based qualitative study (14), rich descriptive data but thin. moderate concerns about adequacy | **Minor concerns:**  Settings cover nursing and medicine. Phenomenon of interest relevant. Concerns about generalisability outside the context studied but finding is not impacted by these concerns | **Moderate confidence** | Moderate methodological and adequacy concerns | |
|  | **-Approachable and flexible**  **(3, 17, 18, 30, 33, 36)** | **Moderate concerns:**  Small sample (3, 30)  Single site(3, 30)  Hawthorne effect(3)  Study based on characteristics drawn from different setting(18)  Response rate due to being mandatory or not receiving results. Inconsistent with voluntary nature of participation(17) | **No or minor concerns:**  Descriptive finding that is well supported by papers contributing to the finding | **Minor concerns:**  1 small sample and 2 larger sample , 1 large delphi ,overall provide good quantity and richness of data supporting the finding | **Minor Concerns**  Settings relevant covering Nursing, Medicine, pharmacy. All in US. Phenomenon of interest relevant. One study used a tool validated in a different setting. Finding not impacted by these concerns | **Moderate confidence** | Moderate methodological concerns | |
|  | **-Enthusiasm for teaching students**  **(5, 6, 14, 15, 31, 37, 40)** | **Moderate concerns**  Small sample(6, 14, 15)  Single site(6, 14, 15, 31)  Large number of criteria leading to difficulty in respondents discriminating between them(5)  Low response rate to Delphi(5)  Self-reported (15) | **No or minor concerns:**  Descriptive finding that is well supported by papers contributing to the finding | **minor concerns**  3 papers had small samples and single sites. Another paper had low Dephi response rate. 2 Qualitative studies provide rich descriptions, but quantity of data thin.  Rigorous literature review provides rich data | **Minor concerns**  Settings relevant covering Nursing, Medicine, pharmacy. Phenomenon of interest relevant Concerns about generalisability outside the context studied, but does not significantly impact the finding. | **Moderate-high confidence** | Moderate methodological concerns | |
|  | **-Open to receiving feedback**  **(5, 17)** | **Moderate concerns:**  Large number of criteria leading to difficulty in respondents discriminating between them(5)  Low response rate to Delphi(5)  Response rate due to being mandatory or not receiving results. Inconsistent with voluntary nature of participation(17) | **No or minor concerns:**  Descriptive finding that is well supported by papers contributing to the finding | **Major concerns:**  Low Delphi response rate despite a wide range of participants, along with the mandatory nature of the response from another paper and the limited number of papers reporting this finding provide major concerns | **Moderate concerns:**  Setting being medicine and pharmacy are relevant. Context is US and Uk. Phenomenon of interest is relevant. Confidence is lowered by the mandatory nature of the responses. | **Low confidence** | Major concerns about adequacy, moderate concerns about relevance and methodology | |
|  |  |  |  |  |  |  |  | |
|  |  |  |  |  |  |  |  | |

1. Boland C, Koval P, Parker M. Determining the utility of a student survey to provide valuable feedback on precepting skills of pharmacy residents. Currents in Pharmacy Teaching and Learning. 2014;6(3):406-11.

2. Bradley H, Cantrell D, Dollahan K, Hall B, Lewis P, Merritt S, et al. Evaluating preceptors: a methodological study. Journal for Nurses in Professional Development. 2015;31(3):164-9.

3. Childs-Kean LM, Ivy DR, Gonzales C, McIntryre W. Development of a tool to evaluate advanced pharmacy practice experience preceptors in the clinical setting at a united states college of pharmacy: A pilot study. Pharmacy Education. 2016;16(1):98-102.

4. Conigliaro RL, Stratton TD. Assessing the quality of clinical teaching: a preliminary study. Med Educ. 2010;44(4):379-86.

5. Cotton P, Sharp D, Howe A, Starkey C, Laue B, Hibble A, et al. Developing a set of quality criteria for community-based medical education in the UK. Education for Primary Care. 2009;20(3):143-51.

6. Cox CD, Peeters MJ, Stanford BL, Seifert CF. Pilot of peer assessment within experiential teaching and learning. Currents in Pharmacy Teaching and Learning. 2013;5(4):311-20.

7. del Bueno DJ, Beay PJ. Evaluation of preceptor competence and cost in an acute care hospital. Journal of nursing staff development : JNSD. 1995;11(2):108-11.

8. Elliot DL, Hickam DH. Medical students' evaluations of their preceptors' teaching in an introductory course. Academic Medicine. 1991;66(4):243-4.

9. Elmore L, Blair M, Edgerton L. Preceptor development strategies used in a mixed academic-community teaching hospital. Currents in Pharmacy Teaching and Learning. 2014;6(1):167-73.

10. Fuller PD, Peters LL, Hoel R, Baldwin JN, Olsen KM. Residency preceptor development and evaluation: A new approach. American Journal of Health-System Pharmacy. 2013;70(18):1605-8.

11. Gueorguieva V, Chang A, Fleming-Carroll B, Breen-Reid KM, Douglas M, Parekh S. Working Toward a Competency-Based Preceptor Development Program. J Contin Educ Nurs. 2016;47(9):427-32.

12. Harris BJ, Butler M, Cardello E, Corelli R, Dahdal W, Gurney M, et al. Report of the 2011-2012 AACP Professional Affairs Committee: Addressing the Teaching Excellence of Volunteer Pharmacy Preceptors. American Journal of Pharmaceutical Education. 2012;76(6):S4.

13. Hartline C. Preceptor selection and evaluation: a tool for educators and managers. J Nurs Staff Dev. 1993;9(4):188-92.

14. Hsu L. An analysis of clinical teacher behaviour in a nursing practicum in Taiwan. J Clin Nurs. 2006;15(5):619-28.

15. Hsu LL, Hsieh SI, Chiu HW, Chen YL. Clinical teaching competence inventory for nursing preceptors: instrument development and testing. Contemp Nurse. 2014;46(2):214-24.

16. Huggett KN, Warrier R, Maio A. Early learner perceptions of the attributes of effective preceptors. Adv Health Sci Educ Theory Pract. 2008;13(5):649-58.

17. Johnson NR, Chen J. Medical student evaluation of teaching quality between obstetrics and gynecology residents and faculty as clinical preceptors in ambulatory gynecology. Am J Obstet Gynecol. 2006;195(5):1479-83.

18. Knisely MR, Fulton JS, Friesth BM. Perceived importance of teaching characteristics in clinical nurse specialist preceptors. Journal of professional nursing : official journal of the American Association of Colleges of Nursing. 2015;31(3):208-14.

19. Lewis BS, Pace WD. Qualitative and quantitative methods for the assessment of clinical preceptors. Fam Med. 1990;22(5):356-60.

20. Lie D, Boker J, Dow E, Murata P, Encinas J, Gutierrez D, et al. Attributes of effective community preceptors for pre-clerkship medical students. Med Teach. 2009;31(3):251-9.

21. Litzelman DK, Stratos GA, Marriott DJ, Skeff KM. Factorial validation of a widely disseminated educational framework for evaluating clinical teachers. Academic Medicine. 1998;73(6):688-95.

22. Melaku T, Srikanth A, Getaye Y, Admasu S, Alkalmi R. Perceptions of pharmacy clerkship students and clinical preceptors regarding preceptors' teaching behaviors at Gondar University in Ethiopia. Journal of educational evaluation for health professions. 2016;13:9.

23. Mintz M, Southern DA, Ghali WA, Ma IW. Validation of the 25-Item Stanford Faculty Development Program Tool on Clinical Teaching Effectiveness. Teaching & Learning in Medicine. 2015;27(2):174-81.

24. Schol S. A multiple-station test of the teaching skills of general practice preceptors in Flanders, Belgium. Academic Medicine. 2001;76(2):176-80.

25. Skeff KM, Skeff KM. Enhancing teaching effectiveness and vitality in the ambulatory setting. Journal of General Internal Medicine. 1988;3(1;2;):S26-S33.

26. Sonthisombat P. Pharmacy student and preceptor perceptions of preceptor teaching behaviors. American Journal of Pharmaceutical Education. 2008;72 (5) (no pagination)(110).

27. Srinivasan M, Li S-TT, Meyers FJ, Pratt DD, Collins JB, Braddock C, et al. “Teaching as a Competency”: Competencies for Medical Educators. Academic Medicine. 2011;86(10):1211-20.

28. Stuart MR, Orzano AJ, Eidus R. Preceptor development in residency training through a faculty facilitator. J. 1980;11(4):591-5.

29. Walter S, Mulherin K, Cox CD. A Preceptor competency framework for pharmacists. Part 2 of a 3-part series. Currents in Pharmacy Teaching and Learning. 2017;10(3):402-10.

30. Al-Arifi MN. Evaluating the preceptor-preceptee relationship among Pharm D students at the King Saud University School of Pharmacy. Saudi Pharmaceutical Journal. 2018;26(6):865-9.

31. Bochenek SH, Fugit AM, Cook AM, Smith Pharm KM. Pharmacy residents' perception of preceptors as role models. American Journal of Health-System Pharmacy. 2016;73(11 Supplement 3):S88-S93.

32. Borimnejad L, Valizadeh S, Rahmani A, Whitehead B, Shahbazi S. Attributes of Iranian new nurse preceptors: A phenomenological study. Nurse Educ Pract. 2018;28:121-6.

33. Brink D, Simpson D, Crouse B, Morzinski J, Bower D, Westra R. Teaching Competencies for Community Preceptors. Fam Med. 2018;50(5):359-63.

34. Ferreira FDC, Dantas FC, Valente GSC. Nurses' knowledge and competencies for preceptorship in the basic health unit. Rev Bras Enferm. 2018;71(Supplement 4):1564-71.

35. Heshmati-Nabavi F, Vanaki Z. Professional approach: the key feature of effective clinical educator in Iran. Nurse Educ Today.30(2):163-8.

36. Jahangiri L, McAndrew M, Muzaffar A, Mucciolo TW. Characteristics of effective clinical teachers identified by dental students: a qualitative study. Eur J Dent Educ.17(1):10-8.

37. L'Ecuyer KM, Hyde MJ, Shatto BJ. Preceptors' Perception of Role Competency. J Contin Educ Nurs. 2018;49(5):233-40.

38. Lee WS, Cholowski K, Williams AK. Nursing students' and clinical educators' perceptions of characteristics of effective clinical educators in an Australian university school of nursing. J Adv Nurs.39(5):412-20.

39. Stenfors-Hayes T, Hult H, Dahlgren LO. What does it mean to be a good teacher and clinical supervisor in medical education? Adv Health Sci Educ Theory Pract.16(2):197-210.

40. Sutkin G, Wagner E, Harris I, Schiffer R. What makes a good clinical teacher in medicine? A review of the literature. Academic Medicine.83(5):452-66.

41. GRIFFITH CHI, GEORGESEN JC, WILSON JF. Six-year Documentation of the Association between Excellent Clinical Teaching and Improved Students' Examination Performances. Academic Medicine. 2000;75(10):S62-S4.
