# Supplementary material for: Measuring and assessing the competencies of preceptors in health professions: a systematic scoping review
Source: BMC Med Educ. 2020 May 24;20:165. doi: 10.1186/s12909-020-02082-9 (PMC7247189; doi:10.1186/s12909-020-02082-9)
Supplement: Supplementary file 4 — Additional file 4. Appendix 4 Risk of Bias appraisal. [file 12909_2020_2082_MOESM4_ESM.docx]

Appendix 4 Risk of Bias appraisal

| CASP ASSESSMENT-Cohort | 1 | | 2 | | 3 | | 4 | 5a | | 5b | | 6a | | 6b | | 9 | 10 | | | 11 |  | |  |  |  |
| --- | --- | --- | --- | --- | --- | --- | --- | --- | --- | --- | --- | --- | --- | --- | --- | --- | --- | --- | --- | --- | --- | --- | --- | --- | --- |
| AUTHOR | Address a clearly focused Issue? | | Cohort recruited in an acceptable way | | Was exposure accurately measure to minimise bias | | Was outcome accurately measure to minimise bias | Identified all confounding factors? | | Taken account of confounding factors in design or analysis | | Follow up of subjects complete enough? | | Follow up of subjects long enough? | | Do you believe results? | Can results be applied to local population? | | | Do results of this study fit with othe available evidence | YES | | CAN'T TELL | NO |  |
| Boland | yes | | can't tell | | can't tell | | no | yes | | can't tell | | can't tell | | can't tell | | yes | yes | | | yes | 3 | | 5 | 1 |  |
| Bradley | yes | | yes | | yes | | yes | yes | | yes | | can't tell | | can't tell | | yes | yes | | | yes | 7 | | 2 | 0 |  |
| Childs-Kean | yes | | yes | | yes | | can't tell | no | | can't tell | | can't tell | | can't tell | | yes | can't tell | | | yes | 4 | | 4 | 1 |  |
| Conigliaro | yes | | yes | | yes | | yes | yes | | can't tell | | can't tell | | can't tell | | yes | yes | | | yes | 6 | | 3 | 0 |  |
| Cox | yes | | no | | can't tell | | can't tell | yes | | no | | can't tell | | yes | | yes | can | | | yes | 4 | | 3 | 2 |  |
| Elliot | can't tell | | yes | | can't tell | | can't tell | can't tell | | can't tell | | can't tell | | can't tell | | can't tell | yes | | | yes | 1 | | 8 | 0 |  |
| Johnson | yes | | yes | | yes | | yes | yes | | can't tell | | can't tell | | can't tell | | yes | yes | | | yes | 6 | | 3 | 0 |  |
| Knisley | yes | | yes | | yes | | yes | no | | no | | can't tell | | can't tell | | yes | yes | | | yes | 5 | | 2 | 2 |  |
| Litzelman | yes | | can't tell | | yes | | yes | yes | | yes | | can't tell | | can't tell | | yes | yes | | | yes | 6 | | 3 | 0 |  |
| Melaku | yes | | yes | | yes | | yes | yes | | yes | | can't tell | | can't tell | | yes | can't tell | | | yes | 7 | | 2 | 0 |  |
| Mintz | yes | | yes | | yes | | yes | yes | | yes | | can't tell | | can't tell | | yes | yes | | | yes | 7 | | 2 | 0 |  |
| Schol | yes | | yes | | yes | | yes | no | | can't tell | | can't tell | | can't tell | | yes | yes | | | yes | 5 | | 3 | 1 |  |
| Sonthisombat | yes | | yes | | yes | | can't tell | can't tell | | can't tell | | can't tell | | can't tell | | yes | can't tell | | | yes | 4 | | 5 | 0 |  |
| Griffith | yes | | yes | | can't tell | | yes | yes | | yes | | yes | | yes | | yes | can't tell | | | can't tell | 8 | | 1 | 0 |  |
| Al-Arifi | yes | | yes | | can't tell | | can't tell | no | | no | | can't tell | | can't tell | | yes | can't tell | | | yes | 3 | | 4 | 2 |  |
| bochenek | yes | | no | | can't tell | | can't tell | no | | no | | can't tell | | can't tell | | yes | can't tell | | | can't tell | 2 | | 4 | 3 |  |
| Lee | yes | | yes | | yes | | yes | can't tell | | can't tell | | can't tell | | can't tell | | yes | yes | | | yes | 5 | | 4 | 0 |  |
| CASP ASSESSMENT- QUAL | | 1 | | 2 | | 3 | | | 4 | | 5 | | 6 | | 7 | | | 8 | 9 | | |  | |  |  |
| AUTHOR | | Clear statement of aims? | | Is qualitative methodology appropriate? | | Was research design appropriate? | | | recruitment strategy appropriate? | | Data collected in a way that addressed the research issue? | | relationship between researcher and participants considered? | | Ethical issues considered? | | | Data analysis rigorous? | Clear statement of findings? | | | YES | | CAN'T TELL | NO |
| COTTON | | yes | | yes | | yes | | | yes | | yes | | can't tell | | yes | | | yes | yes | | | 8 | | 1 | 0 |
| Del Bueno | | yes | | yes | | can't tell | | | yes | | can't tell | | can't tell | | no | | | can't tell | yes | | | 4 | | 4 | 1 |
| Elmore | | yes | | can't tell | | can't tell | | | can't tell | | can't tell | | can't tell | | can't tell | | | can't tell | can't tell | | | 1 | | 8 | 0 |
| Fuller | | yes | | can't tell | | can't tell | | | can't tell | | can't tell | | can't tell | | can't tell | | | can't tell | can't tell | | | 1 | | 8 | 0 |
| Gueorguieva | | yes | | yes | | can't tell | | | can't tell | | can't tell | | can't tell | | no | | | can't tell | yes | | | 3 | | 5 | 1 |
| Hartline | | yes | | can't tell | | yes | | | can't tell | | yes | | can't tell | | can't tell | | | can't tell | no | | | 3 | | 5 | 1 |
| HSU 2006 | | yes | | yes | | yes | | | yes | | yes | | yes | | yes | | | yes | yes | | | 9 | | 0 | 0 |
| HSU 2014 | | yes | | yes | | yes | | | yes | | yes | | can't tell | | yes | | | yes | yes | | | 8 | | 1 | 0 |
| Huggett | | yes | | yes | | yes | | | yes | | yes | | yes | | yes | | | yes | yes | | | 9 | | 0 | 0 |
| Lewis | | yes | | yes | | yes | | | yes | | can't tell | | can't tell | | can't tell | | | yes | yes | | | 6 | | 3 | 0 |
| Lie | | yes | | yes | | yes | | | yes | | yes | | yes | | can't tell | | | yes | yes | | | 8 | | 1 | 0 |
| Skeff | | yes | | can't tell | | can't tell | | | can't tell | | can't tell | | can't tell | | can't tell | | | can't tell | yes | | | 2 | | 7 | 0 |
| Srinivasan | | yes | | yes | | yes | | | yes | | can't tell | | can't tell | | can't tell | | | can't tell | yes | | | 5 | | 4 | 0 |
| Stuart | | yes | | yes | | yes | | | yes | | yes | | yes | | can't tell | | | yes | yes | | | 8 | | 1 | 0 |
| Walter | | yes | | yes | | yes | | | yes | | yes | | yes | | yes | | | can't tell | yes | | | 8 | | 1 | 0 |
| Borimnejad | | yes | | yes | | yes | | | no | | no | | can't tell | | yes | | | can't tell | yes | | | 5 | | 2 | 2 |
| Brink | | yes | | yes | | yes | | | yes | | yes | | can't tell | | can't tell | | | yes | yes | | | 7 | | 2 | 0 |
| Ferreira | | yes | | yes | | yes | | | can't tell | | yes | | can't tell | | yes | | | can't tell | can't tell | | | 5 | | 4 | 0 |
| Hesmati-Nabavi | | yes | | yes | | can't tell | | | no | | yes | | can't tell | | can't tell | | | yes | yes | | | 5 | | 3 | 1 |
| Jahangiri | | yes | | yes | | yes | | | yes | | yes | | can't tell | | can't tell | | | yes | yes | | | 7 | | 2 | 0 |
| L'Ecuyer | | yes | | yes | | yes | | | yes | | yes | | can't tell | | can't tell | | | yes | yes | | | 7 | | 2 | 0 |
| Stenfors-Hayes | | yes | | yes | | yes | | | yes | | yes | | can't tell | | can't tell | | | can't tell | yes | | | 6 | | 3 | 0 |
| Sutkin | | yes | | yess | | yes | | | yes | | yes | | can't tell | | yes | | | yes | yes | | | 7 | | 1 | 0 |
